# A comprehensive stalagmite investigation distinguishing anthropogenic and natural signals in Madagascar between 1680 and 1860

**DOI:** 10.1038/s41598-025-09222-5

**Published:** 2025-07-08

**Authors:** Ny Riavo G. Voarintsoa, Hallie M. Fowler, Thomas J. Lapen, Avotriniaina Z. M. Rakotovao, Ali Raza, Xianglei Li, Hai Cheng

**Affiliations:** 1 Department of Earth and Atmospheric Sciences, University of Houston, Houston, TX USA; 2 Core Laboratories, Reservoir Group, Houston, TX USA; 3 Geological Society of Madagascar, Antananarivo, Madagascar; 4 Mention Bassins Sédimentaires Evolution Conservation, Faculté des Sciences, Université d’Antananarivo, Antananarivo, Madagascar; 5 Institute of Vertebrate Paleontology and Paleoanthropology, Chinese Academy of Sciences, Beijing, China; 6 Institute of Global Environmental Change, Xi’an Jiaotong University, Xi’an, China; 7 State Key Laboratory of Loess and Quaternary Geology, Institute of Earth Environment, Chinese Academy of Sciences, Xi’an, 710061 China

**Keywords:** Madagascar, O and C isotopes, Redox conditions, Anthropogenic traces, Elemental composition, Microstratigraphic log, Biogeochemistry, Environmental sciences

## Abstract

Disentangling anthropogenic from natural induced changes is difficult, but crucial to accurately assess the specific environmental impacts of humans’ actions versus climate in the paleoclimate records. Here we combine a new set of proxies, including stable isotopes, element concentrations (mainly Mg, Sr, and U), and detailed mineralogy to better distinguish the impacts of these two factors in the stalagmite records. We studied the period between 1680 and 1860 CE because of the known historical events in Madagascar history (e.g., western colonization and the growth of Malagasy kingdom). This is an ideal period to study given these known events. Our data suggest that redox conditions during alternating wet and dry conditions is revealed by U/Ca covarying with δ^18^O, but extreme climatic conditions may reverse that relationship. We also found an Mg/Ca increase combined with a decrease in δ^13^C starting ca. 1820 CE that suggest anthropogenic imprints associated with local burning in Madagascar. This new multiproxy combination, paired with a thorough understanding of Madagascar history over this time allowed us to distinguish anthropogenic versus natural–induced signals in Madagascar stalagmite. This unique and novel pairing of proxies can be used to understand and predict human and natural induced changes in similar settings.

**Supplementary Information:**

The online version contains supplementary material available at 10.1038/s41598-025-09222-5.

## Introduction

Anthropogenic activities, such as the Suess effect, urbanization, and land use changes, have been identified in several stalagmite records worldwide^1–5^. In Madagascar, the widespread anthropogenic impacts on the landscape of Madagascar and its environment over the past centuries has sparked interest in several scientific disciplines (e.g.,^6–9^), the idea of which made disentangling the impacts of natural climate and human activities in the paleoclimate records challenging and yet unresolved. For example, the rapid landscape transformation inferred from a large stalagmite δ^13^C shift of ~ 10–12‰ in Anjohibe Cave, in northwest Madagascar, to reflect anthropogenic activities^10–13^, has been challenged by a recent cave survey that revealed comparable isotopic range of ~ 10‰ explaining natural processes within the same cave^14^. Organic geochemistry also supports that those δ^13^C changes do not reflect shift to modern landscape^15^. These challenges suggest that much remains to be learned about human imprints and natural climate change signals in Madagascar paleoclimate records.

**Fig. 1:**
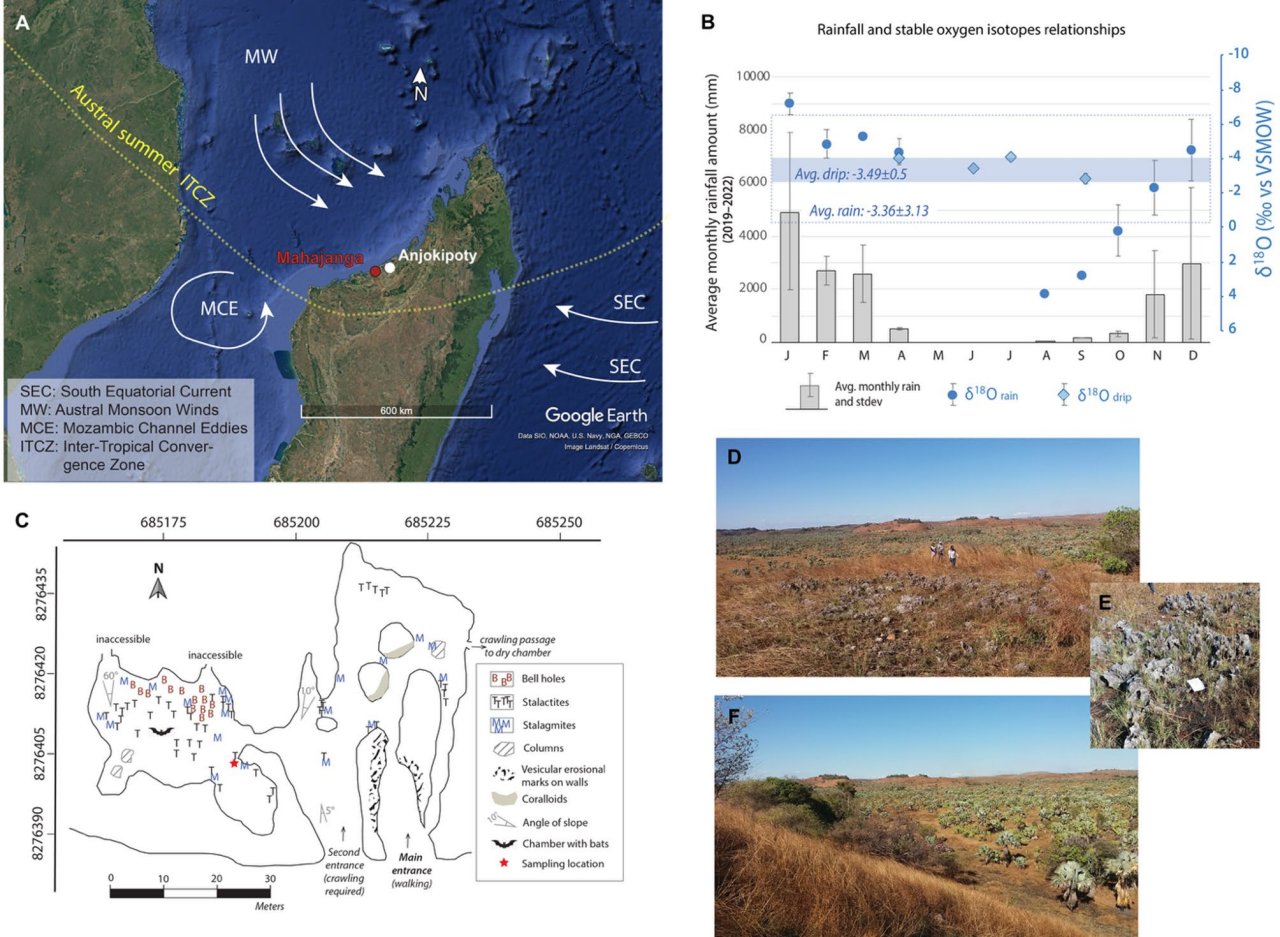
Anjokipoty Cave settings. (**A**) Regional map overlayed by the major ocean currents and the southern position of the ITCZ in austral summer and the geographic location of Anjokipoty Cave and the main city, Mahajanga. The background map is a Google Earth Imagery, Data SIO, NOAA, U.S. Navy, NGA, GEBCO produced using Google Earth Pro 7.3.6.10201 (64-bit). (**B**) Measured mean rainfall amount from Mahajanga and the corresponding mean water δ^18^O values (circle^32^) along with the measured Anjokipoty δ^18^O drip water values (diamond, this study). (**C**) Detailed map of the principal chamber of Anjokipoty Cave that was mapped in 2018–2019 and showing the sampling location (red star). The cave was georeferenced in ArcGIS 10.5 using the Oblique Mercator Laborde projection^33^. (**D**) Photo of the bedrock exposure and vegetation cover right above the cave. (**E**) Close-up photo of the exposed bedrock and less developed soil and grasses taken during the 2018 field investigation (the white square in the center is a 15 × 20 cm notebook). (**F**) C4 savanna grasses typical of the cave surroundings at higher elevation with endemic satra palms in valleys. Additional cave setting information is available in the Supplementary.

This paper combines a new set of proxies, including δ^18^O, δ^13^C, Mg/Ca, Sr/Ca, U/Ca, and mineralogy, to best identify natural and human-induced signals in a stalagmite from Anjokipoty Cave, in northwestern Madagascar (Fig. 1a), between 1680 and 1860 CE, where rainfall is seasonal (Fig. 1b). Stalagmites are excellent paleoclimate archivers as their growth can be accurately constrained using U-Th chemistry and they can produce high-resolution paleoclimate dataset (e.g.,^16–18^). The period between 1680 and 1860 CE is a time of significant changes in Madagascar history, if not only mentioning the pre-colonial time and the growth of Madagascar Kingdoms (e.g.,^19–24^), when agricultural related activities, such as slash and burns, began to develop. Thus, it is an ideal interval to assess both human and natural induced signals in Madagascar paleoclimate records.

### Cave site setting

Anjokipoty Cave belongs to a tertiary limestone, called Narinda South karst, which is part of the southern part of a westward gently dipping (3–5°) Eocene limestone layer of the post-Karroo formation within the sedimentary basin of Mahajanga^25^^,^^26^. This Eocene limestone forms the Narinda karst^27^ (see Supplementary S1). Based on a geochemical survey at the limestone stratigraphic outcrop at Mariarano River^28^, the karst lithology varies from very pure, porous (15.8% porosity), and quite homogeneous limestone (~ 99.1% of CaCO_3_ with only 0.9% of residual clays) at the base, to less porous (5%) and dolomitized lithology (~ 62.1% CaCO_3_ and 30.2% MgCO_3_) in the upper part, where dolines are typically found.

Regional altitude in the surrounding area of Anjokipoty varies between 40 and 70 m above sea level, and the cave itself is at about 63–69 m in altitude (Supplementary S1). Anjokipoty Cave develops under a small hill, the vegetation above which is sparse with patches of savanna grasses. Vegetation in its immediate surrounding, i.e., in the surrounding valleys, is a tropical savanna dominated landscape decorated with palm trees (Fig. 1d–f).

The cave is small with less than a kilometer walkable and accessible passages. The chamber internal height ranges between 3 and 9 m, and the overburden is thin (~ 1–3 m). Stalagmite MAJ-1 was collected from a fifteen by thirty-meter chamber, a section of which is occupied by bats (Fig. 1c). There are a few evidence of modern human occupations (e.g., remains of tissue fabrics, broken pieces of clay pots and glass, bottle caps, and glass bottles) that we noticed during our field expedition.

For logistics reason, the cave atmosphere was only monitored for a short period (Supplementary S2). Despite the limited cave atmosphere monitoring dataset, the available data remains informative, and they suggest that Anjokipoty is a well-ventilated cave, showing a clear diurnal variation in temperature and pCO_2_. These variations may be associated with the shallowness of the cave and its openness to the surrounding environment. Similar diurnal pattern was observed in a larger nearby Anjohibe Cave, where cave atmospheric seasonality was also reported^29^. With the sensitivity of Anjokipoty Cave’s atmosphere to external daily climatic conditions, it is possible that the cave atmosphere also exhibit seasonal variations, as in Anjohibe Cave.

Rainfall in the region is dependent on the austral migration of the Inter-Tropical Convergence Zone (ITZC) with distinct winter and summer seasons^30,31^. Warm and rainy monsoonal seasons are experienced between November and March, when the ITCZ moves south, and cool and dry winter seasons between April and October, when the ITCZ moves north with a dominant easterly dry trade wind^32^ (Fig. 1a). Ground monitoring of modern rainfall performed between October 2019 and December 2022 in Mahajanga, the main region where the cave belongs, suggests that monthly rainfall varied between 10 and 2166 mm, and confirms that rainfall δ^18^O closely reflect the amount effect, with higher values in drier months and lower values in wetter months^32^. With detailed isotopic analyses using d-excess and ^17^O_excess_, the data suggest that drier months (with < 1000 mm rainfall) are kinetically affected by subcloud evaporation compared to wetter months (with rainfall > 1000 mm), where isotopic equilibrium is attained. These variations are closely tied to the latitudinal migration of the ITCZ and the dominant trade wind that brings moisture to the region. Cave drip waters were also collected in July and September of 2018 and April and June of 2019 (Supplementary S2, Table S1), when roads to the caves were accessible. The averaged drip water δ^18^O values have smaller variability (− 3.49 ± 0.5‰, vs. VSMOW, this study) and represent the mean annual rainfall δ^18^O values (− 3.36 ± 3.13‰, vs. VSMOW^32^) in Mahajanga (Fig. 1b).

## Results

### Stalagmite MAJ-1 description

Stalagmite MAJ-1 is approximately 176 mm tall and 70 mm wide at its base narrowing to about 15–20 mm at its top. It grew as a single stalagmite from 176 to 100 mm (distance from the top), with a slightly curved growth axis, and splits into two stalagmites (twin A and twin B, see Figure S4 in Supplementary S3) at the upper 100 mm. The growth axis of twin A is nearly vertical, whereas that of twin B is slightly tilted. The twin A of stalagmite MAJ-1 seems to grow faster compared to its contemporaneous twin B. Given the narrowness of the stalagmite growing up, sampling enough powders for U-Th was only possible for twin A. The principle of lateral continuity and superposition were then carefully applied to drill for U-Th trenches and to establish the chronology of the stalagmite (Figure S3). Like other stalagmites from tropical regions (e.g.,^12,34–36^), Stalagmite MAJ-1 does not have clear mm-thick laminations, except at a few intervals and at its flank.

### Stalagmite chronology

**Fig. 2:**
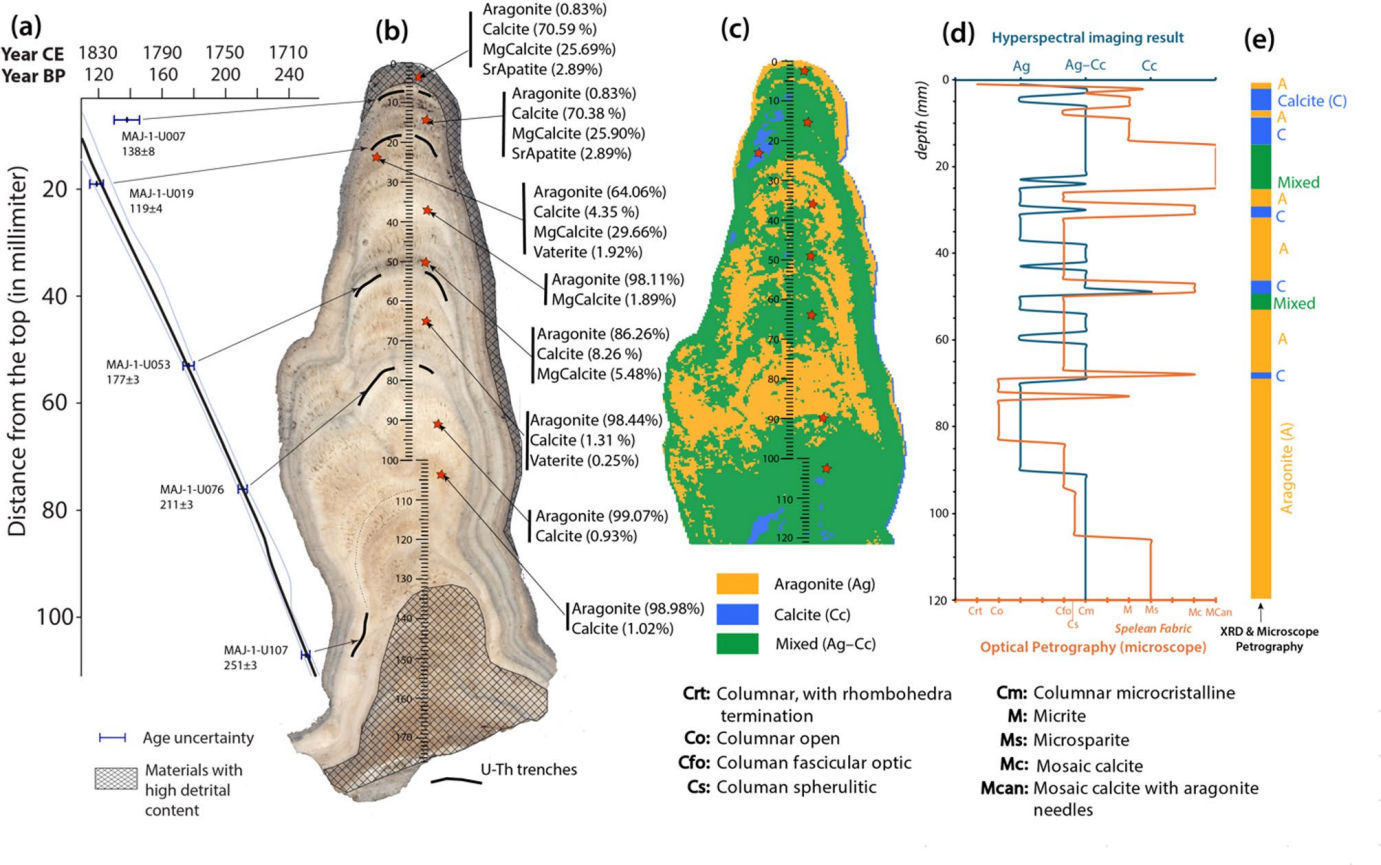
Stalagmite MAJ-1 with its reconstructed chronology and detailed mineralogy. (**a**) StalAge model^37^ of the upper 116 mm of Stalagmite MAJ-1. The thick black line represents the best fit, and the grey lines are the reconstructed uncertainty after the Monte Carlo simulations (see supplementary S4 for details on the Monte Carlo simulation). (**b**) Image of Stalagmite MAJ-1 with a longitudinal scale showing the trenches locations for U-Th chemistry, and the corresponding U-Th dates in BP. Materials with high detrital contents are highlighted with cross-fill pattern. The locations for XRD analyses are indicated with red stars along with the XRD results. XRD patterns and the phase percentages results are provided in the Supplementary. (**c**) Preliminary mineralogy map developed using Hyperspectral imaging, overlain with the location of XRD samples. (**d**) Mineralogy and microstratigraphic log obtained from HSI (blue) and optical petrographic observations using thin sections (orange), respectively. The spelean fabric code was adopted from the acronyms proposed by Frisia^38^. (**e**) Summary of calcite–aragonite classification combining XRD and optical microscopy observations.

The studied 116 mm portion of Stalagmite MAJ-1 from Anjokipoty Cave, within which five U-Th dates were analyzed, has an excellent age constrain. The age uncertainties (2 σ) are exceptionally small, i.e., ranging between 3 and 4 years (Table S2). The sample at 7 mm from the top is an exception, with slightly larger uncertainty (8 years) and appears 20 years older due to natural detrital contamination. Despite the small sample number, the short time interval and the very small dating errors are sufficient to produce reliable models for its chronology. The StalAge modelled chronology (see Fig. 2a, Supplementary S4.1 and Figure S5–S9) suggests that Stalagmite MAJ–1 grew continuously for about ~ 174 years at a rate of 0.7 mm.yr^−1^, yielding a sub-annual resolution for each isotopic sampling.

### Mineralogy and spelean fabric

**Fig. 3:**
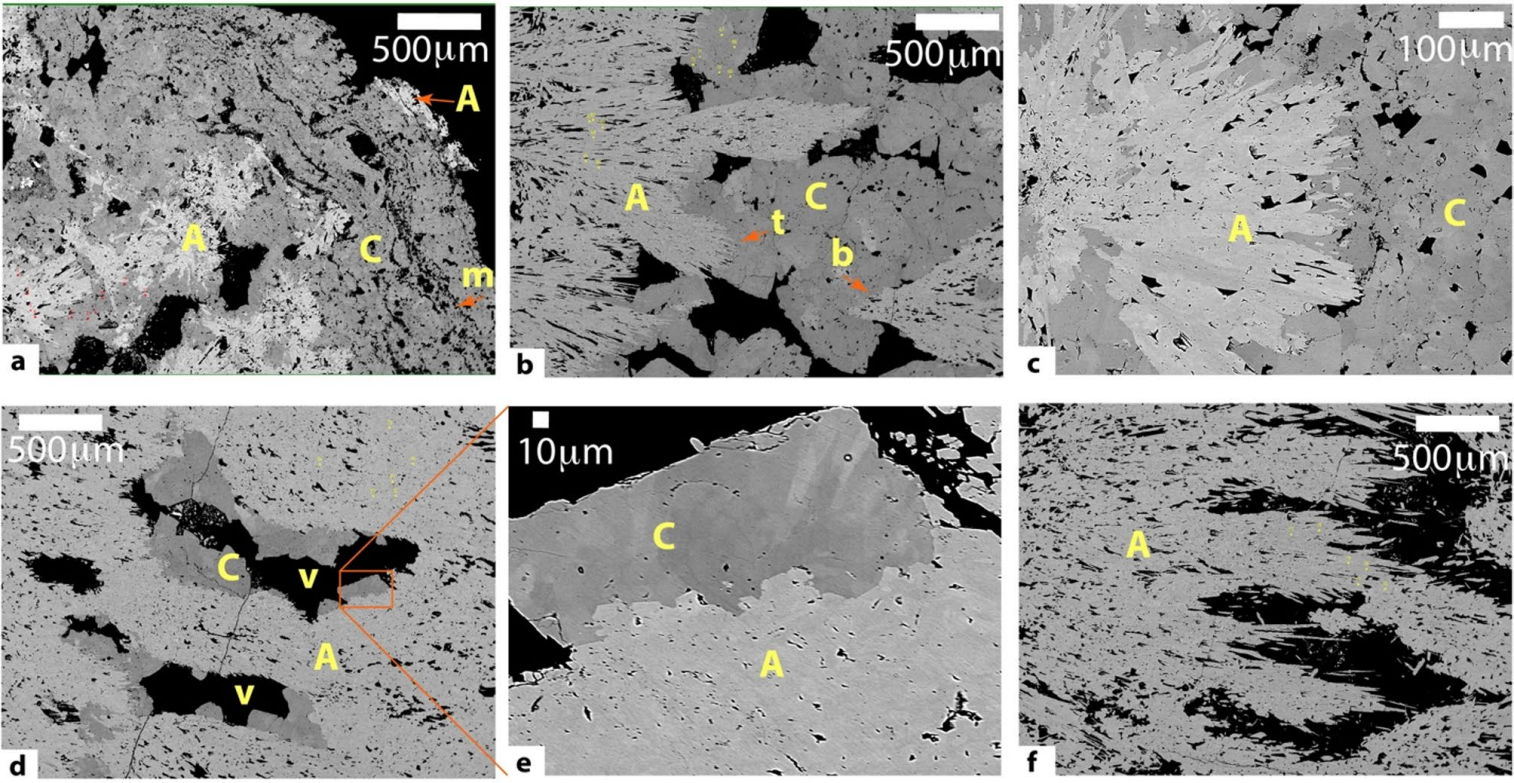
A series of images showing the various mineralogy and their interrelationship. (**a**) Image of the uppermost part of Stalagmite MAJ-1 that is composed of aragonite needles (A) and micritic (m) calcite (C). These micritic fabric (m) are bacterial mats. (**b**) Image of the transition layer between aragonite (A) and calcite (C) where calcite shows mosaic fabric and aragonite with a fibrous termination (t). Note the bottom (**b**) of a new fascicular aragonite growing above the mosaic calcite. (**c**) Higher resolution of the aragonite–calcite relationship, showing the acicular termination of aragonite and the mosaic fabric of calcite. (**d**) Image showing evidence of porosity or voids (v) between crystals of aragonite (A) where crystals of calcite (C) start to grow. (**e**) Close-up of the calcite identified in Fig. 3d highlighting the typical rhombic termination. (**f**) Image of an unaltered fascicular/fibrous aragonite with the primary porosity (black). See Fig. 2 for the summary mineralogy.

Stalagmite MAJ-1 is polymineralic (Figs. 2 and 3, see also Figures S10–S14), with aragonite and a small percent of calcite (< 1.5%) dominating the lower part (between 54 and 120 mm from the top, i.e., before 1775 CE). Petrographic microscope observations of that lower portion show mineral fabric variations from microsparite, columnar spherulitic, columnar fascicular optic, to columnar open. A thin layer (~ 1 mm) of mosaic calcite was found at 68 mm (around 1750 CE). Above 54 mm, the mineralogy is more variable, and it consists of an alternating layer of calcite/magnesium calcite, aragonite, and mixed mineralogy. X-ray diffraction (XRD) reveals an increase in Mg calcite (> 25%) starting at around 25 mm from the top (~ 1812 Year CE), and only minor trace between 30 and 53 mm. XRD also reveals a minor trace of Sr apatite (~ 3%) at the upper ~ 20 mm of Stalagmite MAJ-1, which was identified in back-scattered electron, BSE (Figure S14). While cross-checking between the XRD results and petrographic microscopy, the Mg calcite identified by the XRD method are mostly composed of micritic fabric and/or mosaic calcite surrounding aragonite needles. Both aragonite and calcite are primary deposits (Fig. 3), and the calcite deposition are found to fill gaps and voids between the aragonite crystals (Fig. 3d–e).

### Stable isotopes

**Fig. 4:**
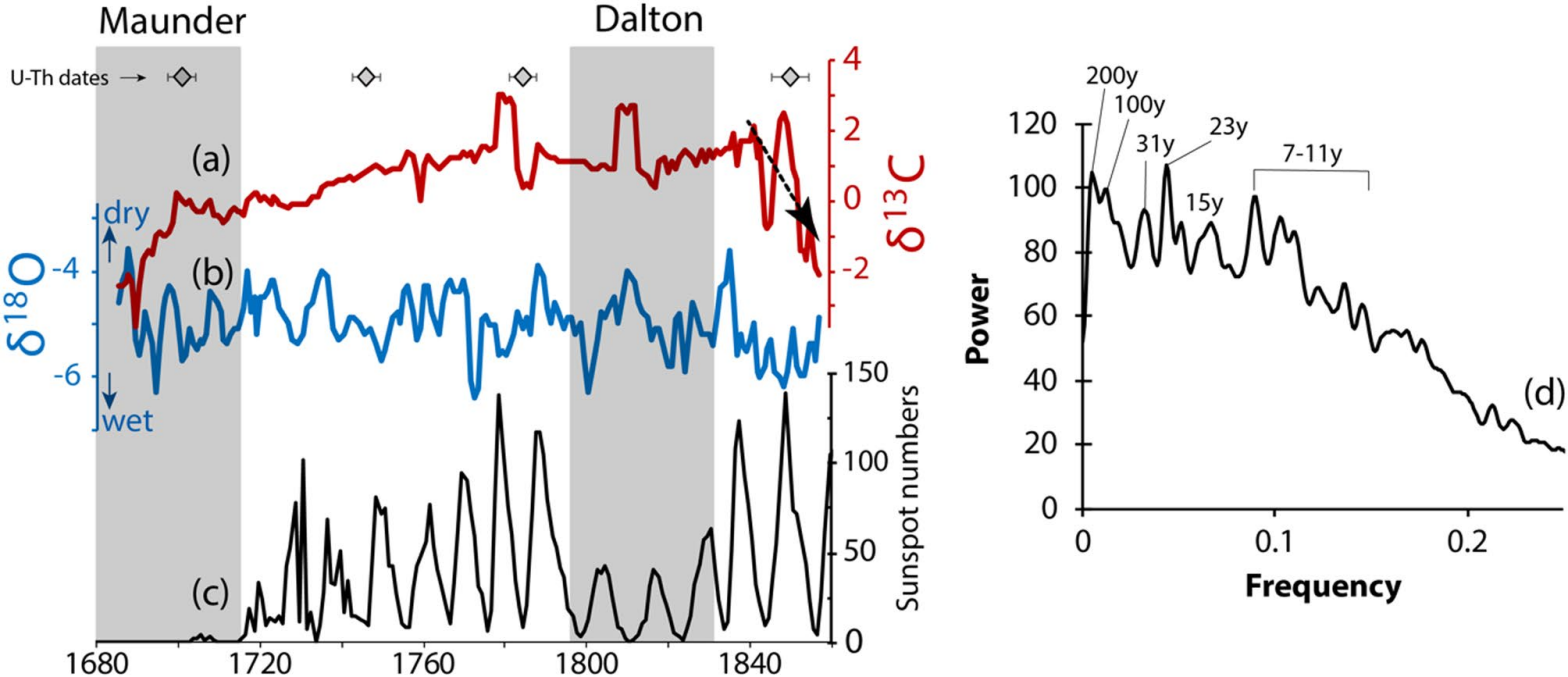
Stalagmite MAJ-1 stable isotope datasets. (**a**) Stable isotopes of carbon time series. (**b**) Stable isotopes of oxygen time series. (**c**) Sunspot number time series. (**d**) Spectral analysis results for δ^18^O dataset. The sunspot numbers are annual means of the corrected international/Zurich/Wolf number defined by Lockwood et al.^41,42^.

There are no major temporal shifts in the magnitude and there is no strong correlation between δ^18^O_c_ and δ^13^C_c_ (Fig. 3a,b). For the carbon isotopes, δ^13^C_c_ values range from − 3.6 to + 3.0‰ VPDB (avg. + 0.5‰), with more positive values, except before ~ 1700CE (around the Maunder minimum) and after 1825CE where values are noticeably trending towards more negative values than the entire record (at the end of the Dalton minimum). For the oxygen isotopes, δ^18^O_c_ values range from − 6.4 to − 3.6‰ VPDB (avg. − 5.0‰), with a noticeable periodicity (Fig. 3d). A spectral analysis, using the Bartlet power spectrum and the Blackman-Tuckey Method, on these stable isotope datasets, using QAnalyseriesWASM^39^^,^^40^ revealed a strong 7–10 periodicity starting ca. 1770CE, and a multidecadal periodicity, with the 20–30 years cycle observed since 1710CE until the end of the record (Fig. 4d). The decadal periodicity is comparable to the Sun’s 11-year solar cycle, and most of the higher δ^18^O values coincides with higher sunspot numbers.

### Elemental composition

**Fig. 5:**
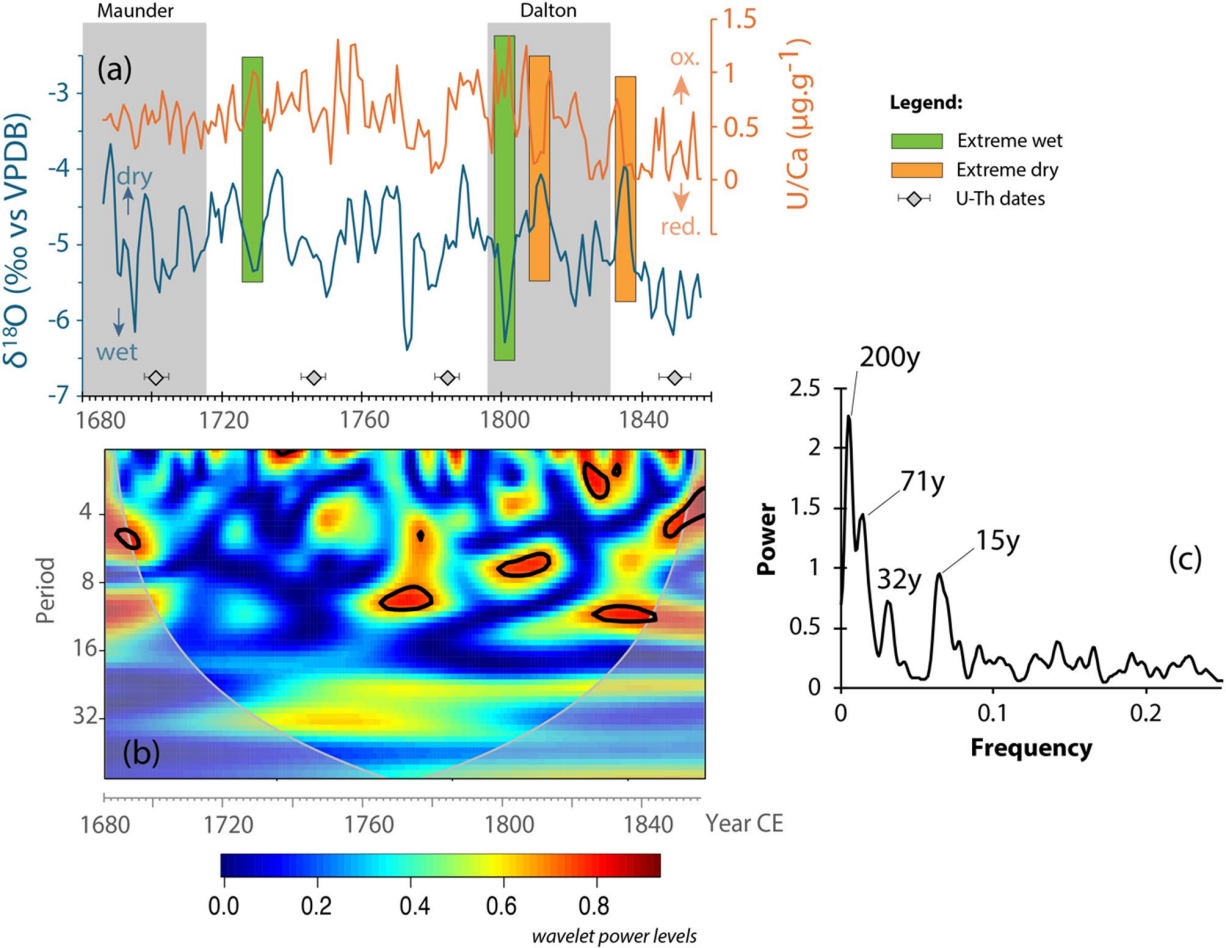
Uranium and δ^18^O covariations. (**a**) Time series of δ^18^O (blue) and U/Ca ratio (orange) along with their environmental implications (for discussion). Note that the U/Ca data were smoothed to match with the temporal resolution of δ^18^O. (**b**) Wavelet coherence plot that was run on the evenly smoothed U/Ca and δ^18^O dataset. (**c**) Spectral analysis profile for U/Ca.

One of the most striking findings from the elemental datasets is the U/Ca variability. The U/Ca ratio surprisingly shares similar pattern with δ^18^O_c_ throughout the interval studied, with values ranging from 0.002 to 1.99 µg g^−1^ (Fig. 5a). Exceptions were found at ~ 1720, 1800, 1805, and 1837 CE, where those patterns are reversed, i.e., low δ^18^O_c_ but high U/Ca and high δ^18^O_c_ but low U/Ca (Fig. 5a). A Pearson’s product-moment correlation test on all U/Ca data (smoothed to match the isotopic resolution) and on the δ^18^O_c_ data suggests a statistically significant weak to moderate positive correlation (r = 0.23, *p* value = 0.005). This covariation is confirmed by a cross-wavelet coherence plot, which additionally highlights the temporal covariance on a decadal/multidecadal scale, and spectral analysis further suggests a 15- and 30-year periodicity (Fig. 5c), identical to what was observed with δ^18^O_c_ (Fig. 4c). These multi-decadal periodicities were identified in several stalagmites from this region of Madagascar, as reviewed in Voarintsoa^43^.

We also found that magnesium and strontium show opposite behavioral pattern with lower Mg/Ca but higher Sr/Ca values at the lower part of stalagmite MAJ–1, between ~ 25 mm and 116 mm (i.e., before ~ 1810 CE), and a noticeable increase in Mg/Ca and more variable Sr/Ca ratios at the upper part (i.e., above ~ 25 mm). The Mg/Ca ratios range between 0.003 and 12.82 µg g^−1^ and the Sr/Ca ratio ranges between 0.04 and 30.3 mg g^−1^. Petrographic observations and XRD results combine to suggest that at the lower portion, between 25 and 116 mm, of stalagmite MAJ-1, the higher Sr concentrations are closely associated with columnar fabric aragonite minerals. At the upper 25 mm, however, the high Sr content appears to be attributed to Sr-bearing apatite and the increase in Mg to reflect the presence of magnesium-bearing calcite (Fig. 2b).

## Discussion

### Environmental significance for stable isotope variations

The stable isotope composition of the spelean carbonates reflects the stable isotopic composition of the parent solution and the dissolved inorganic carbon (DIC) from which they precipitate, their relationship is predicted by the isotopic fractionation factor between the carbonate and the precipitating solution/DIC^32^. A simplified review is provided in the supplementary S7. The isotopic composition of such solution may vary on long or short-term depending on external and internal factors (see for example Fig. 1b; Table S2).

**Fig. 6:**
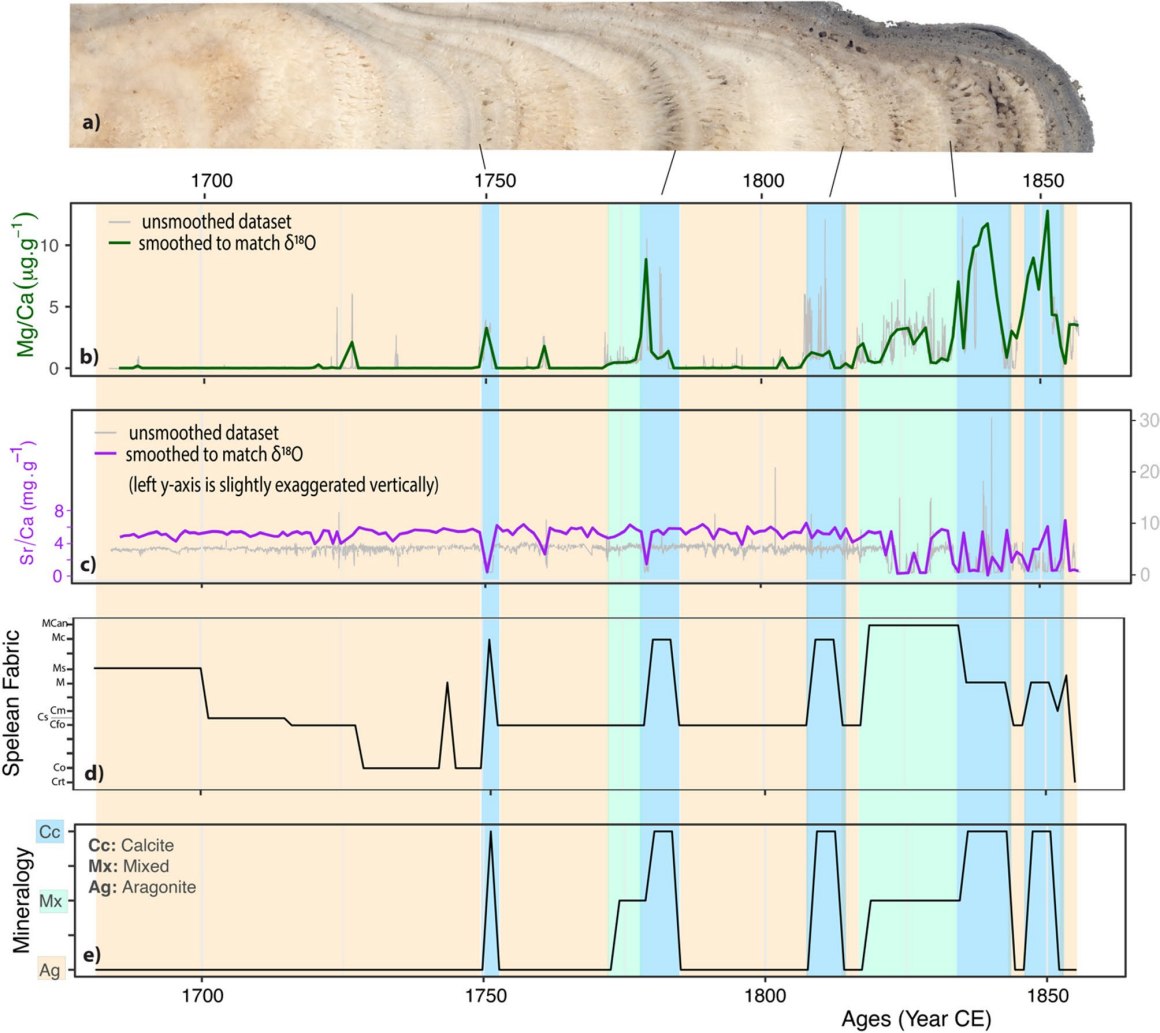
Mg/Ca, Sr/Ca, spelean fabric, and mineralogy variations in Stalagmite MAJ-1. (**a**) Partial image of Stalagmite MAJ-1 showing true color variations. (**b**) Mg/Ca ratio time series in ppm. (**c**) Sr/Ca ratio time series in %. (**d**) Converted temporal log of spelean microfabric using the code developed by Frisia^38^. See Fig. 2 for the explanations of the fabric codes. (**e**) Summary of the overall mineralogical variations based on XRD and detailed petrography, which has been color-coded to create the coloring bars in this figure and in Fig. 7

For δ^13^C_c_, the changes may be closely linked to the fast degassing and CaCO_3_ precipitation rate at the apex of Stalagmite MAJ-1. This is for example the case for the fast calcite nucleation mediated by bacterial and microbial activities found in Australia, leading to the formation of micrite and microsparite and an increase in δ^13^C_c_ by a magnitude of + 6.5‰ (e.g.,^44^). This could also explain the presence of micritic fabric and high δ^13^C_c_ in Stalagmite MAJ-1, which are documented around 1770 and 1810 CE (Figs. 4 and 6), where δ^13^C_c_ is high (~ 3‰). It is also important to note that Anjokipoty Cave is a shallow and a small cave, suggesting a short DIC residence time, and the vegetation cover is dominated by C_4_ grasses, the photosynthetic pathway of which preferentially incorporates ^13^C, and the isotopic composition of which is transferred into the precipitated carbonate. Under warmer and drier climatic conditions, as reflected by the deposition of aragonite, the seeping water may have equilibrated with the soil CO_2_, which is relatively enriched in ^13^C (i.e., reflecting the C_4_ vegetation it supports), and then precipitate aragonite or calcite inside the voids and pores prior to reaching the apex of the stalagmite. These processes could combine to enrich the residual DIC with the heavier ^13^C, and hence the overall high δ^13^C_c_ values of Stalagmite MAJ-1. Lastly, the decreasing trend post-1820 CE can suggest anthropogenic traces that reflect local burning. Details about anthropogenic signals in Stalagmite MAJ-1 are discussed further below.

The δ^18^O in northwestern Madagascar has been interpreted to closely reflect the amount effect because of the summer monsoonal regime of rainfall linked to the latitudinal migration of the Intertropical Convergence Zone (ITCZ) (e.g.,^13,45,46^). The amount effect is a relationship between rainfall amount and δ^18^O, with more negative δ^18^O values during rainier months (e.g.,^47–50^). The recent rainfall monitoring performed in Mahajanga^32^ confirmed this amount effect relationship between rainfall and δ^18^O. Average drip water δ^18^O values from Anjokipoty Cave (− 3.49 ± 0.5‰, vs. VSMOW; Table S2) reflect the mean annual δ^18^O of the rainfall (− 3.36 ± 3.13‰, vs. VSMOW^32^; Fig. 1b). If we use the cave analog isotopic fractionation equation^51^, 1000ln^18^α = 16.516 ± 1.267 × 10^3^/T(K) − 26.141 ± 4.356, and the updated worldwide isotopic fractionation equation^32^, 1000ln^18^α = 16.89 ± 0.62 × 10^3^/T(K)  − 27.41 ± 2.14, that was initially defined by Tremaine et al.^52^, along with the measured cave temperature (22 °C) during the monitoring period in winter (Supplementary S2), the predicted calcite δ^18^O_c_ values vary between − 4.10‰ and − 3.85‰VPDB, respectively (Table S2). These values can change if the initial water δ^18^O_w_ changes, i.e., if rainfall amount is high, the value would become more negative, reflecting the amount effect. If using the regional mean annual temperature (27°C) in the region between 2018 and 2019^29^, the predicted calcite δ^18^O_c_ values are − 5.04 and − 4.80‰, vs. VPDB, respectively. The central tendency of δ^18^O_c_ of Stalagmite MAJ-1 varies around − 5.0 ± 0.6‰, with a minimum and maximum value of − 6.4 and − 3.6‰, respectively. These values generally agree with the predicted δ^18^O_c_ above, confirming the usefulness of stalagmite δ^18^O_c_ as an excellent paleohydrology proxy. The δ^18^O_c_ fluctuations in the record can primarily reflect the amount effect associated with the ITCZ. We also found that the wavelet coherence plot along with the spectral analysis on the δ^18^O time series highlight a significant decadal/multidecadal periodicity (mainly 7–11years, 15 years, 23 years, 31years), with significant interrelation with U/Ca at the 15 and 32-year periodicity (Figs. 4d and 5c). These periodicities have been identified in several stalagmites from this region of Madagascar (as reviewed in Voarintsoa^43^), and were inferred to potentially reflect anomalous year with tropical cyclones^29^. These anomalous rainfall years can be associated with sea surface temperature (SST) anomalies in the immediate Indian Ocean and the Agulhas Current (e.g.,^53–55^), teleconnected with other climatic phenomena in the Indo-Pacific region. A time series correlation using coral records from Ifaty, in southwestern Madagascar revealed similar multidecadal periodicities of SST anomalies in the Aghulas Current (Figure S15), which Zinke et al.^55^ associated with the SST variations in the western and northeastern Pacific, a pattern similar to the Pacific Decadal Oscillation^56^. The comparative records suggest that a positive temperature anomaly in the Aghulas region is associated with drier climates in northwestern Madagascar. Finally, the decadal periodicities (7–15 yrs) seem to tie with changes in solar activities, as shown in Fig. 4c, where higher sunspot numbers are associated with high δ^18^O, which reflects drier climatic conditions. If these relationships are combined, we can summarize that the decadal change in solar activity affects SST and rainfall in Madagascar, and they are well preserved in Stalagmite MAJ-1 during the studied period.

### Uranium as a redox proxy in stalagmites

The relatively high concentration of uranium in Stalagmite MAJ-1, converted from the raw CPS values using the matrix-matched calibration approach (see Methods and Figure S19), can be primarily explained by the dominant aragonite mineralogy that composes it. Despite their scarcity, aragonite speleothems have become valuable in paleoclimate reconstruction because of their high uranium concentration (e.g.,^57,58^), allowing for high precision U–Th dating^59^. Uranium is commonly incorporated as UO_2_(CO_3_)_3_ and readily substitutes Ca within the aragonite crystal structure compared to calcite^60^, with a distribution coefficient of uranium above 1 in aragonite, and below that in calcite^58,61^. Jamieson et al.^58^ specifically used shifts in uranium concentration across primary calcite-to-aragonite transitions in speleothems to calculate the distribution coefficient of uranium in aragonitic speleothems and suggested prior aragonite precipitation as a proxy for rainfall. A basic review on uranium chemistry is provided in the supplementary S9.

While the source, the variations of uranium, and the drivers of such variations in cave systems have been debated and proposed in the literature (e.g.,^58,62–66^), some of which to reflect limestone dissolution (e.g.,^67^) and bacterial reduction (e.g.,^62^), we know that uranium has unique elemental and ionic properties^68^. As reviewed in the supplementary document, U concentration in groundwater closely reflects redox conditions. Higher U contents are associated with reduced conditions, due to the high organic content reducing U^6+^ to U^4+^ (e.g.^62^). This results in low U concentration in the stalagmites under normal rainfall year conditions. In contrast, lower U concentration in the epikarst is associated with oxidizing conditions, due to the strong mobility of UO_2_^2+^. This mobile UO_2_^2+^ percolates downward to the cave and precipitate with the speleothem carbonates, resulting in high U concentration in stalagmites. This red-ox condition can be the geochemical process resulting from the open-closed systems in the epikarst above the cave, reflecting the water–air-rock interaction during wet (closed, reduced conditions) and dry (open, oxidizing) climatic conditions. Due to the inhibiting capacity of CO_3_^2−^ that prevents U adsorption to organic matter or iron oxides, its abundance in groundwater during limestone dissolution can closely control the mobility of uranium in natural waters, and vice versa.

Exceptions are, however, found under extremely wet and under extremely dry conditions. For example, we found that around ~ 1720, 1800, 1805, and 1837 CE, the wet-dry/red-ox patterns are reversed, i.e., low δ^18^O_c_ but high U/Ca and high δ^18^O_c_ but low U/Ca (Fig. 5a). Under extreme wet conditions, with more limestone dissolution and thus an increase in CO_3_^2-^ in the epikarst water, the adsorbed uranium in the epikarst may have been washed by faster water percolating down to the cave. This would increase the amount of uranium carried down to the cave, resulting in high U/Ca in the stalagmite. Under extreme dry conditions, however, there may be very little water, so that everything would precipitates into carbonates in the epikarst during prior aragonite precipitation (aka PAP^58^) and thus resulting in low U/Ca in the stalagmite.

While we used the U/Ca ratio as a tracer for this redox condition/process in the epikarst and at the apex of the stalagmite, the data suggest that its covariation with δ^18^O_c_ (Fig. 5), a widely used proxy for rainfall proxy in Madagascar, potentially suggest red-ox evidence under varying climatic conditions. Under normal wet conditions, as shown by the more negative δ^18^O_c_ values, i.e., under closed water–air-rock interaction in a reducing environment, U/Ca in Stalagmite MAJ-1 is low because the high adsorption capacity of organic matter in the epikarst. The opposite behavior is observed under normal dry conditions, as shown by the high δ^18^O_c_, i.e., under open water–air-rock interaction in an oxidizing environment, where UO_2_^2+^ is mobilized to combine with CO_3_^2−^. The multidecadal periodicity (15 and 32–year) identified in both δ^18^O_c_ and U/Ca also tend to suggest that redox conditions in Anjokipoty cave are tightly controlled by climate variations, which in turns is influenced by solar activity. Close attention to the pattern also helps identify extreme climatic conditions, as they may seldom reverse that wet-dry/red-ox relationship.

### Climatic and non-climatic significance of mineral variations

The formation of aragonite and calcite has been used as a tool indicator for changes in climate, with aragonite indicating drier climate and calcite of wetter climate^46,69,70^. Others have attributed changes in polymorphs to reflect changes in drip water chemistry, such as pH of the drip water, saturation index for calcite, and specifically Mg/Ca ratio of the parent water^71–75^. Experimental studies under constrained laboratory conditions demonstrated that the formation of aragonite over calcite is a function of the growth temperature and of the water chemistry, specifically the Mg/Ca ratio, of the parent solution from which the carbonate precipitates^76–78^. Referring to Fig. 1 of Balthasar and Cusack^76^, in which the proportion of aragonite versus calcite is detailed under varying laboratory conditions, and assuming that the mean cave temperature in Anjokipoty Cave (~ 21 °C) has not changed during the time period being studied, pure calcite could form with Mg/Ca < 1, pure aragonite would form with higher Mg/Ca ratio (> 3), and a mixture of both between 1 and 3. If this assumption is true, the parent solution chemistry leading to the precipitation of calcite and/or aragonite is a pure reflection of the drip water chemistry, which in turn represents the host bedrock geochemistry, and it could be associated with the alternating layer of limestone and dolomite as described in the cave setting section. However, aragonite layers in Stalagmite MAJ-1 have a very low to undetectable Mg content (Fig. 6). This obviously leaves a major gap in our understanding of the aragonite–calcite deposition in this cave.

Without changing the initial Mg/Ca ratio of the parent solution, a parent solution without Mg or with low Mg/Ca ratio (≤ 1) can fully precipitate aragonite at higher temperature, starting at ≥ 25 °C^76–78^. For example, with an initial Mg/Ca of 1, a mixture of calcite and aragonite could start forming at ≥ 15 °C^76^, and a 10 °C temperature increase would result in near 100% precipitation of aragonite^77^. If such experimental results can be transferred to interpret aragonite precipitation in Anjokipoty Cave, and if climate is the main driver, aragonite deposition could remain an indicator of warm and dry climate, and calcite an indicator of cool and wetter climate^46,69,70^.

It is also common to assume that diagenesis would transform aragonite into calcite for stalagmites that are composed of both polymorphs, especially when calcite is found enrobing aragonite or aragonite appearing as a ghost inside calcite in optical microscopy^38,79^. Thin section microscopy shows that the spelean fabric of the mixed mineralogy, with 64% aragonite, between 15 and 25 mm from the top of the stalagmite is composed of a needle of aragonite within a mosaic of calcite, and much of the calcite depositing at the uppermost 15 mm of the stalagmite is composed majorly of micritic/microsparitic fabric. A cross-verification of this calcite-aragonite relationship using SEM BSE images suggests that the aragonite needles and the mosaic calcite crystals are instead primary (Fig. 3). The calcite minerals are found to fill gaps and voids between the aragonite crystal (see specifically Fig. 3d–e), and the micritic/microsparitic fabric seems to result from high nucleation rates mediated by microbial and bacterial mineralization. We specifically found a trace of microorganism in the upper part of Stalagmite MAJ-1 (Figure S16). The fast nucleation rate and the high microbial activities could have eased the incorporation of Mg into the calcite lattice to precipitate Mg calcite, as shown by the increase in the % of Mg calcite per XRD results. The presence of a fully preserved gastropod shell (Figure S17) in the upper layer of stalagmite MAJ-1 and the lack of age reversal (except the upper layer with high detrital content, Table S2) additionally suggest against diagenetic transformation of aragonite to calcite.

Finally, the presence of Sr-bearing apatite (Ca_5_(PO_4_)_3_OH) in the upper 15 mm of Stalagmite MAJ-1, revealed by XRD and BSE image (Figs. 2 and S14) potentially reflects a high biogenic input, as additionally suggested by the presence of micro-organism trace and gastropods (Figure S16–S17). This high biogenic activity could be primarily associated with the presence of bat colony in the cave (Fig. 1b) and secondarily with the leaching product of the cave overburden. The chemical reaction between the main calcium carbonate minerals and the leachates from guano deposits, which contains phosphoric acid, can form carbonate-apatite precipitates. They appear as a dark grey/dark brown encrustation and the color change is obvious in the upper 10 mm of Stalagmite MAJ-1 (Figs. 2a and 6a), due to the high organic content. The occurrence of apatite mineral in a fossil or modern bat guano deposit in cave environment is well documented, for example in the Prehistoric Cave at Azé, in France^80^, in the “dry” Cioclovina Cave, in the Şureanu Mountains of Romania^81^, and in many European caves^82^. The conditions that favor precipitation of calcium carbonate also favor formation of carbonate-apatite, including pH (which can be as low as 7) and a slightly reducing environmental conditions. Overall, the presence of the gastropod shells and microorganism (Figure S16–S17) in the upper part of Stalagmite MAJ-1 is a good indication of an increase in biological activity in the cave, the timing of which is estimated to begin around 1820–1830 CE.

### Mg/Ca and Sr/Ca variations to indicate mineralogy instead of PCP

Mg and Sr are commonly used as a proxy for PCP or prior calcite precipitation (e.g.^58,75,83–86^) because of their strong partitioning behavior and abundance in karst environment. Recent cave monitoring in Anjohibe Cave, which develops within the same Narinda Karst as Anjokipoty Cave, have also shown evidence of PCP in drip water chemistry using the conventional Mg/Ca and Sr/Ca ratios, with pronounced PCP signals at the winter-summer transition^29^.

While these former findings inspire us to find similar signals in Anjokipoty Cave, we instead found that Mg/Ca and Sr/Ca variations in Stalagmite MAJ-1 do not seem to always represent PCP. A cross-comparison and cross-evaluation of these elements with the stalagmite petrography (using microscope), along with EPMA data and BSE images suggest that Mg and Sr concentrations in Stalagmite MAJ-1 are primarily an excellent predictor for mineralogy, i.e., for carbonate polymorph (Fig. 6). The data suggest that Sr is mainly associated with aragonite and Mg is mainly associated with calcite, especially Mg calcite. Both calcite and aragonite preserve their primary features in Stalagmite MAJ-1 (Figs. 3, S12 and S13). Those relationships have been identified in other stalagmites, such as Stalagmite AB2 from the nearby Anjohibe Cave, where Sr/Ca ratios vary in parallel with the % aragonite in the sample^87^. In stalagmites HK1 and HK3 from Grotte Prison de Chien, in Morocco, Wassenburg et al.^75^ also reported that primary calcite layers contain up to 1.9 mol% MgCO_3_ in HK1 and 3.25% in HK3, with clear elemental variations across a lateral calcite and aragonite transition, i.e., variations from the center towards the flank of the stalagmite. Their data show (see their Fig. 5 for Stalagmite HK1 and Fig. 6 for Stalagmite HK3) that Mg concentrations gradually increase at the aragonite-calcite lateral transition, with a pronounced shift at the layer-bounding surfaces between the two polymorphs by a factor of 60–90. Similarly, Sr concentrations gradually decrease at the aragonite–calcite transition, with a pronounced shift at the layer-bounding surfaces between the two polymorphs by a factor of 7–9.

### Biogenic and anthropogenic evidence after 1820CE

There is a pronounced Mg/Ca ratio increase in Stalagmite MAJ-1 starting ca. 1820 CE (Fig. 5a). Within the sample, this increase is associated with calcite, the fabric of which varies from mosaic to micrite, but the highest values are associated with non-diagenetic micrite and microsparitic fabric (see discussion about Mineralogy). Experimental studies demonstrate that the formation of micrite requires a very high number of nuclei, high supersaturation and/or the presence of organic compounds^38,88^. Biotic interference has been observed in terrestrial carbonates, such as laminar calcretes and tufas, where micrites formation is intervened by cyanobacteria ^44,89^. It was also observed in cave moonmilk and other calcareous tufa where micrites are influenced by biogenic activities^90,91^. In Anjokipoty cave, the increase in biogenic activities is confirmed by the precipitation of Sr-Apatite (Figure S14), the preservation of microscopic organic traces (Figure S16), and the preservation of gastropod shells (Figures S17). The presence of micrite has been reported in stalagmites from Nullarbor, Australia, to dramatically increase δ^13^C by about 6.5 ‰ (i.e., ranging from − 10.5 to − 4.5 ‰^44^), and this can explain the δ^13^C_c_ peaks in Stalagmite MAJ-1, observed at ~ 1770 and 1810CE.

**Fig. 7:**
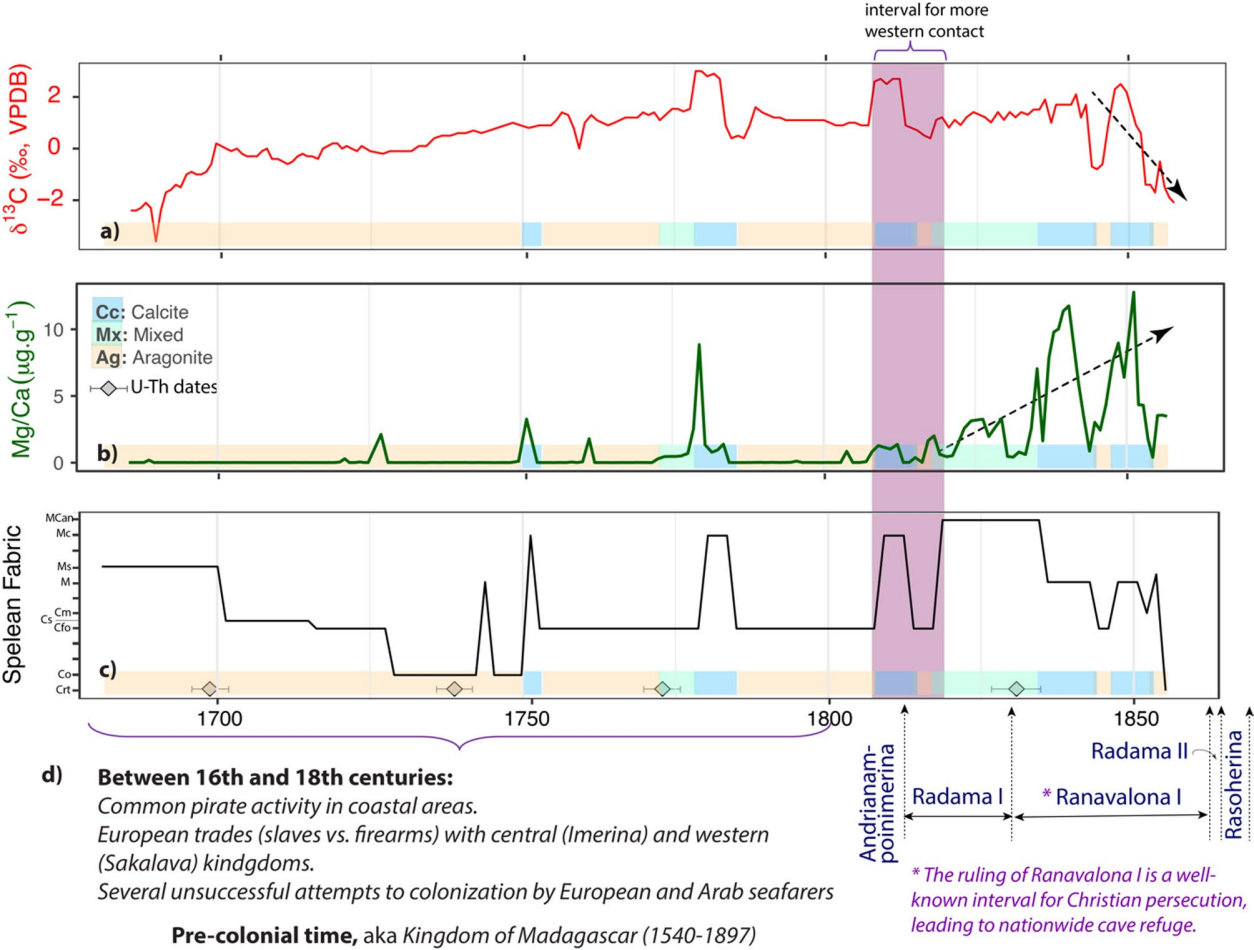
Anthropogenic proxies and historical summaries in Madagascar between the 16th and the nineteenth century. (**a**) Stable isotopes of carbon time series highlighting a decreasing trend due to local burning towards the end of the record. (**b**) Mg/Ca ratio time series in ppm series highlighting an increasing trend starting ca. 1820 CE. (**c**) Spelean microfabric temporal log. See Fig. 2 for the explanations of the fabric codes. (**d**) Summary of Madagascar pre-colonial times, highlighting with an asterisk the period when cave inhabitation during the ruling of Ranavalona I. Those historical summaries were obtained from various sources (e.g.,^19–24^).

While the increase in Mg/Ca ratio in Stalagmite MAJ-1 is concurrent with the precipitation of micritic calcite and an increase in biogenic activities and inputs, it is also important to understand possible external sources for magnesium, such as anthropogenic inputs as the period of speleothem growth coincides with Madagascar pre-colonial times, known as the Kingdom of Madagascar (Fig. 7), with the pivotal development of dynasties and the creation of Menabe and Boina in western Madagascar, and a well-documented historical record of western contact, such as the dominance of Christian missionary education (e.g.,^19–24^). It is specifically aligned with the Kingdom of Queen Ranavalona I, during which Christian persecutions led to human refuging into caves and population migration and re-settlement. The history of human settlement or the associated foreign colonization in Madagascar is often associated with local burning, such as slash and burn agriculture (e.g.,^7,92^) or setting up wood fire while refuging inside the cave. The burning could locally produce more CO_2_ enriched in light carbon (^12^C) in the atmosphere, leading to a decrease in the overall δ^13^C values, similar to the well-known ‘Suess effect’^93–95^, and this appeared to be preserved in the period after 1820 CE (indicated with an arrow in Fig. 7a).

With the small U-Th dating errors (3–4 years), the timing of Mg/Ca increase aligns well with the well-documented human activity in Madagascar (Fig. 7). Combined with the local burning discussed above, the increase in magnesium content in Stalagmite MAJ-1 also indicates an anthropogenic origin, either through slash and burn activities or through cave refugia or temporary inhabitation of humans using wood torch. Mg is a significant component of wood ash^96^ and its incorporation into calcite must have been strongly mediated by the microbial activity and high nucleation rate during micrite formation. The increase in Mg/Ca ratio post 1820CE, with marked change in coloration to brownish and gray (Figs. 6a and Figure S17), could thus be the combination of increased microbial activities and leaching of Mg from magnesium bearing minerals from wood ash that is being washed into the cave by the percolating waters following slash and burn activities. It could also be the result of aerosol Mg from wood torch, while people used caves for temporary residence during this pre-colonial times/fierce persecution of Christians or a target location for hunting^21^. Human frequent presence into the caves could enhance the rate of bacterial and microbial activities in the cave, and hence the dominance of micritic deposit at the upper portion of Stalagmite MAJ-1.

## Conclusions

Our research revealed evidence of both naturally induced changes, mainly redox conditions, and anthropogenic imprints between 1680 and 1860 CE. First, redox conditions are highlighted by a statistically significant covariation between δ^18^O and U/Ca, well-pronounced at the decadal and multidecadal scale (15 and 30-year periodicity), and potentially associated with solar cycles and SST anomalies in the immediate Indian Ocean and the Agulhas Current, which is teleconnected with other climatic phenomena in the Indo-Pacific region. Periods with more rainfall can enhance the growth of biomass activity, leading to reduced conditions in the cave overburden. Since uranium ions are adsorbed on organic matter, these reducing conditions result in low U/Ca in the stalagmite record. In contrast, drier climate favors an open water–rock interaction into more oxidizing conditions, favoring U mobilization as uranyl ion, the latter percolating down to the cave and incorporates into the crystal lattice of aragonite, inducing a higher U/Ca. However, extreme climatic conditions may reverse these relationships due to prior aragonite precipitation. Second, anthropogenic traces are best reflected in the Mg/Ca increase, a change in coloration from white to dark gray, and a decrease in δ^13^C due to local burning, the resulting isotopic responses are similar to the Suess effect, after 1820 CE, the timing of which coincides with a well-documented human activity, specifically a linkage with slash-and-burn activities and cave inhabitation.

The detailed documentation of mineral fabric has also made paleoclimate and paleoenvironment interpretation more robust. We found that elemental variations (mainly Mg/Ca and Sr/Ca) are better proxies for mineralogy than for climate, although both are indirectly linked. The absence of Mg in spelean aragonite opens a venue to investigate in detail the conditions of its formation, especially under changing temperature. Lastly, detailed documentation of mineralogy and mineral fabric helped usunderstand the influence of calcite and aragonite precipitation dynamics and biogenic matter incorporation on the isotopic and elemental composition of speleothems. We will further test and consolidate this innovative multiproxy combination on other stalagmites with similar age.

## Methods

### Mineralogy identification

Three complementary methods were primarily used to recognize mineral variability within Stalagmite MAJ-1: (1) Hyperspectral Imaging, (2) X-ray Diffraction, and (3) petrographic analyses using thin sections.

Hyperspectral Imaging (HSI), a relatively new method in the speleothem field uses two SpecIm hyperspectral cameras (Spectral Imaging Ltd., Finland). These cameras are designed to acquire images in wider spectral range covering visible to near-infra-red (VNIR) and short-wave infrared (SWIR) region (see details in Raza et al.^97^). Prior to the imaging, the halved portion of Stalagmite MAJ-1 was polished to remove surface irregularities. Images were taken within a spectral range between 400 and 2500 nm, and raw images were treated using the ENVI 5.7 software (NV5 Geospatial Solutions, Inc.). To cross-check the accuracy of the data acquired from those cameras, point spectral analysis was done using an ASD FieldSpec Pro spectroradiometer. This spectroradiometer was calibrated with Spectralon^98^. A decision tree classifier, which is a built-in algorithm within the ENVI 5.7 software, was then applied to automize mineral classification following the method developed in Raza et al.^97^, using the calcite index developed by Ninomiya^99^. This non-destructive method allows to quickly map mineral variations at the exposed surface of the sample. With the high primary porosity of Stalagmite MAJ-1 (~ 10%, estimated based on the blue epoxy distribution that we used to impregnate the thick section slabs), HSI was only able to provide a general, less accurate, distribution of mineralogy (Fig. 2b). This is because light easily disperses within the porous surface, and this can potentially affect the reflectance spectra^100^. After developing the microfabric log of the spelean fabric using thin section following Frisia^38^ (see further below), we found some mismatch between the HSI results and the thin section observations. We then used these data to select spelean layers for further X-Ray Diffraction analyses.

A total of eight spelean layers were sampled to extract powders (about 20–30 mg) at the PaleoGeochem Lab using a handheld dental drill (see Fig. 2b,c, red stars, for the location of these samples), each stored in a 5 mL centrifuge vials for subsequent XRD analyses. The powders were then mounted on a zero-diffraction plate, introduced into the Rigaku SmartLab X-ray Diffractometer, then scanned between 20° and 60° (2-theta diffraction angle) for 10 min at 40 kV and 44 mA using the Copper K-α radiation. XRD analyses were performed at the Texas Center for Superconductivity at the University of Houston. The XRD patterns were then uploaded in Profex, an open-source software to identify phases and to quantify their percentage in each sample^101^.

The combination of petrographic observations (using thin sections) with the HSI and XRD datasets is a comprehensive approach to accurately define mineralogy variations as well as mineral fabric changes throughout the stalagmites. Frisia^38^ specifically states that a systematic documentation of calcite fabrics in stalagmites can increase robustness in paleoclimate reconstructions that use geochemical proxies. Two oversized and one regular thin section were prepared at the Quality Thin Sections in Arizona to cover the upper 116 mm of Stalagmite MAJ-1, with which a microstratigraphic log of spelean fabrics was developed. Those thin sections were examined at the Department of Earth and Atmospheric Sciences of the University of Houston, using a Nikon Eclipse LV100 polarized microscope. A suite of thin section microphotograph was continuously taken to create a mosaic per thin section (Figs. 2; S10). A scale was added for each of these mosaics, in reference to photograph resolution and the thin section dimension, and a fabric log was correspondingly developed along the distance from the top of the stalagmite (Fig. 2d). We then applied the mineral fabric code proposed by Frisia^38^ to systematically document the calcite and aragonite fabrics. Finally, we combined the developed microstratigraphy log, the XRD data, and HSI results to conclude on the mineral composition of Stalagmite MAJ-1 (Fig. 2e).

Backscatter electron (BSE) microscopy images that were obtained during the use of Electron Probe Microanalyzer for the internal matrix-matched calibration (see further below and Supplementary S10) was used as a complementary dataset to the main mineralogy method above. The images were used to further resolve crystalline and diagenetic structure in Stalagmite MAJ-1. It was also used to investigate transitions between carbonate polymorphs.

### IRMS for stable isotopes

The methods we used for stable isotope measurements are identical to those described in Voarintsoa et al.^102^, which follow the analytical methods described in Paul and Skrzypek^103^. A total of 177 powdered carbonate samples from the upper 116 mm of Stalagmite MAJ-1 were discretely and consistently milled using a Micro-Cut dental drill at sub-millimeter intervals (i.e., sub-annual). About 80–100 μg of that carbonate powders was weighted on a Sartorius microbalance and transferred to a 4.5 mL round bottom Labco® exetainers. The samples were flushed with Helium and acidified with H_3_PO_4_ at 50 °C and analyzed using the GasBench II technique in continuous flow IRMS at the Alabama Stable Isotope Laboratory of the University of Alabama. All isotopic ratios were expressed in the delta notation relative to Vienna Pee Dee Belemnites (VPDB).

With the non-monomineralic nature of Stalagmite MAJ-1, it is necessary to correct the δ^18^O results for phosphoric acid fractionation effects between aragonite and calcite. The calcite and aragonite acid fractionations factors are 1.00937 and 1.00967, respectively at the acidification temperature of 50°C^104,105^. This translates into a correction values of + 0.31‰ for layers containing aragonite. Mixed mineralogy layers were corrected based on the % of aragonite based on methods outlined in Voarintsoa et al.^78^.

It is also known that aragonite and calcite, when grown at the same environment, shows a clear polymorph fractionation with aragonite preferentially incorporating heavier carbon isotopes than calcite. In a synthetic carbonate precipitation experiment, Romanek et al.^106^ found that aragonite–bicarbonate enrichment factors average 2.7 ± 0.6‰ and those of calcite average 1.0 ± 0.2‰, suggesting a 1.7 ± 0.4 fractionation between aragonite and calcite. To account for this mineralogical bias in incorporating heavier isotopes, all aragonite samples were corrected by –1.7‰ and mixed mineralogy were corrected based on the % of aragonite in the sample, as has been done in Sletten et al.^70^ and Voarintsoa et al.^102^.

### LA-ICP-MS for elemental composition

Thick sections (~ 100 μm) were prepared and polished at TPS Enterprises LLC in Bellaire, Texas, for laser ablation analyses. Samples were mounted on a blue epoxy to easily identify pores and voids in the samples while performing the laser measurements. The LA-ICP-MS measurements were done at the Department of Earth and Atmospheric Sciences of the University of Houston using a Jena PlasmaQuant Quadrupole ICP-MS coupled with a Teledyne PhotonMachines Excite 193 nm laser system. The laser ablation of a continuous transects were performed at a rate of 30 μm/s with a 50 μm in diameter circular beam and a laser firing rate of 15 Hz. A pre-ablation line scan was also done prior to each LA-ICP-MS measurement to even the sampling line topography and to remove potential surface contaminations. Two parallel line scans were run then averaged to amend for possible lateral variability in trace elements, which can be partially controlled by their site preference during crystal growth (e.g.,^107^). LA-ICP-MS data reduction was performed using the technique detailed in^108^.

Since the raw LA-ICP-MS on Stalagmite MAJ-1 datasets were time resolved and are expressed in counts per seconds (CPS), the final elemental concentration (in wt proportions) of element X was calculated using an internal matrix-matched calibration approach (Eq. 1). To do this, we performed spot analyses by electron probe microanalysis (EPMA) (see supplementary file, Section S11) to obtain accurate elemental concentration of the internal reference materials, that include calcite and aragonite. Backscatter electron (BSE) microscopy was used to identify the sampling locations within the thick sections (Figure S18 and Section S11). We performed spot analyses on thirteen aragonite minerals and on eight calcite minerals that appear clean and do not show porosity or other obvious signs of alterations, such as micro corrosions or intracrystalline porosity. The spot analyses performed on each mineral represent an average of six individual spot measurements within the matrix (Figure S18), thus a total of 78 measurements for aragonite and 48 for calcite. We run spot analyses on the LA-ICP-MS closer to these EPMA spots to pre-calibrate the line scans, the final concentration of which were calculated using Eq. (1).

1 $$\left[\text{X}\right]=\left[{\text{E}}_{std}\right]\times {\text{I}}_{\text{spot}}\times {[\text{E}}_{\text{spot}}]\times {\text{I}}_{\text{line}}$$

In the equation above, [X] is the final trace element concentration that was calculated using the internal matrix-matched calibration.$\left[{\text{E}}_{std}\right]$ is the concentration of the internal standard (e.g., Ca determined by EPMA) in either calcite or aragonite. ${\text{I}}_{\text{spot}}$ is the background subtracted intensity ratio of element X to the internal standard (Ca) in the unknown sample from the LA-ICP-MS spot measurements. [${\text{E}}_{\text{spot}}]$ is the concentration ratio of element X to the internal standard in the reference material, either calcite or aragonite from the EPMA. ${\text{I}}_{\text{line}}$ is the background subtracted intensity ratio of element X to the internal standard (Ca) in the unknown sample from the LA-ICP-MS line scan measurements.

Using the summary mineralogy (Fig. 2e), the final elemental concentration [X] was respectively calculated to matrix-match the polymorph occurrence in the sample. In other words, calcite samples were calculated using the EPMA measured values for calcite and aragonite samples were calculated using the EPMA measured values for aragonite. Finally, we verified the elemental concentration conversion of the LA-ICP-MS dataset by referring to the available uranium-238 measured during U-Th chemistry (Table S1, Section S1) using the multi-collector inductively coupled plasma mass spectrometry (MC-ICP-MS, Table S2, Figure S19).

The LA-ICP-MS concentration and the measured MC-ICP-MS shows a good concentration agreement (Figure S19). The temporal resolution of the LA-ICP-MS data is also very high, so the data were smoothed to match the temporal resolution of the stable isotopes for further statistical analysis.

### U-Th dating

About 50 to 250 mg of CaCO_3_ powder was extracted from each trench for U-Th dating. The chemical procedures are like those described in Edwards et al.^109^ and Shen et al.^110^ when separating uranium and thorium. U-Th measurements were performed on the multi-collector inductively coupled plasma mass spectrometry (MC-ICP-MS) at the Stable Isotopes Laboratory of Xi’an, in Jiaotong, China. Instrument details are provided in Cheng et al.^111^. Corrected ^230^Th ages assume an initial ^230^Th/^232^Th atomic ratio of 4.4 ± 2.2 × 10^−6^, which is the ratio for “bulk earth” or crustal material at secular equilibrium with a ^232^Th/^238^U value of 3.8. The radiometric data are reported as years BP, where BP = before present, and present is AD 1950. Results from U-Th chemistry are shown in Table S2. Note that the uppermost part (at 7 mm) has a very high detrital content, so despite the high uranium concentration, the resulting age appears older that the samples below it. Using the StalAge, this sample was excluded from building the final age model.

Individual isotope and element sampling locations, as well as the log for mineralogy, were recorded as a function of the distance from the top (dft), expressed in millimeter, of the stalagmite, and with the Monte Carlo simulation, these depth values were converted into individual age (Figures S5–S9). The age model created from StalAge was then used to convert the distance from the top to an age, with a 2-sigma error, that anchors the stable isotope data, the element concentration data, and the mineralogy and mineral fabric variations throughout the interval studied (Fig. 2a). The ages were originally reported as Before Present (with present = 1950), and then converted to Common Era (CE), following equation Year CE = 1950 – Year BP, to better represent the modern time coverage.

## Electronic supplementary material

Below is the link to the electronic supplementary material.

Supplementary Material 1

Supplementary Material 2

## Data Availability

All data associated with this work are available in the supplementary file, and they can also be requested to the corresponding author.
