# Supplementary material for: A comprehensive stalagmite investigation distinguishing anthropogenic and natural signals in Madagascar between 1680 and 1860
Source: Sci Rep. 2025 Jul 8;15:24362. doi: 10.1038/s41598-025-09222-5 (PMC12238641; doi:10.1038/s41598-025-09222-5)
Supplement: Supplementary file 2 — Supplementary Material 2 [file 41598_2025_9222_MOESM2_ESM.docx]

**Supplementary document for**

**A comprehensive stalagmite investigation distinguishing anthropogenic and natural signals in Madagascar between 1680 and 1860**

*Ny Riavo G. Voarintsoa^1*^, Hallie M. Fowler^1–2^, Thomas J. Lapen^1^,* Avotriniaina Z. M. Rakotovao^3–4^, *Ali Raza ^1^, Xianglei Li^5^, and Hai Cheng^6–7^*

**^1^** Department of Earth and Atmospheric Sciences University of Houston, Houston, Texas, United States

**^2^** Core Laboratories, Reservoir Group, Houston, Texas, United States

**^3^** Geological Society of Madagascar, Antananarivo, Madagascar

**^4^** Mention Bassins Sédimentaires Evolution Conservation, Faculté des Sciences, Université d’Antananarivo, Madagascar

**^5^** Institute of Vertebrate Paleontology and Paleoanthropology, Chinese Academy of Sciences, Beijing, China

**^6^** Institute of Global Environmental Change, Xi’an Jiaotong University, Xi'an, China

**^7^** State Key Laboratory of Loess and Quaternary Geology, Institute of Earth Environment, Chinese Academy of Sciences, Xi’an 710061, China

*Corresponding author: nyriavo.voarintsoa@gmail.com; ngvoarin@central.uh.edu

# S1. Cave Settings

Anjokipoty Cave, located in northwestern Madagascar (Figure 1), owes its name to its very small and shallow size, as the word “kipoty” in Malagasy means small. The summed length of all walkable and accessible passages, based on our survey and cave mapping in 2018 and 2019, does not exceed one kilometer, and the largest chamber, in which Stalagmite MAJ-1 was collected, measures ~15 m and ~30 m in width and length, respectively (Figure 1). The chamber internal height ranges between 3 and 9 m, and the overburden is thin (~1–3m).


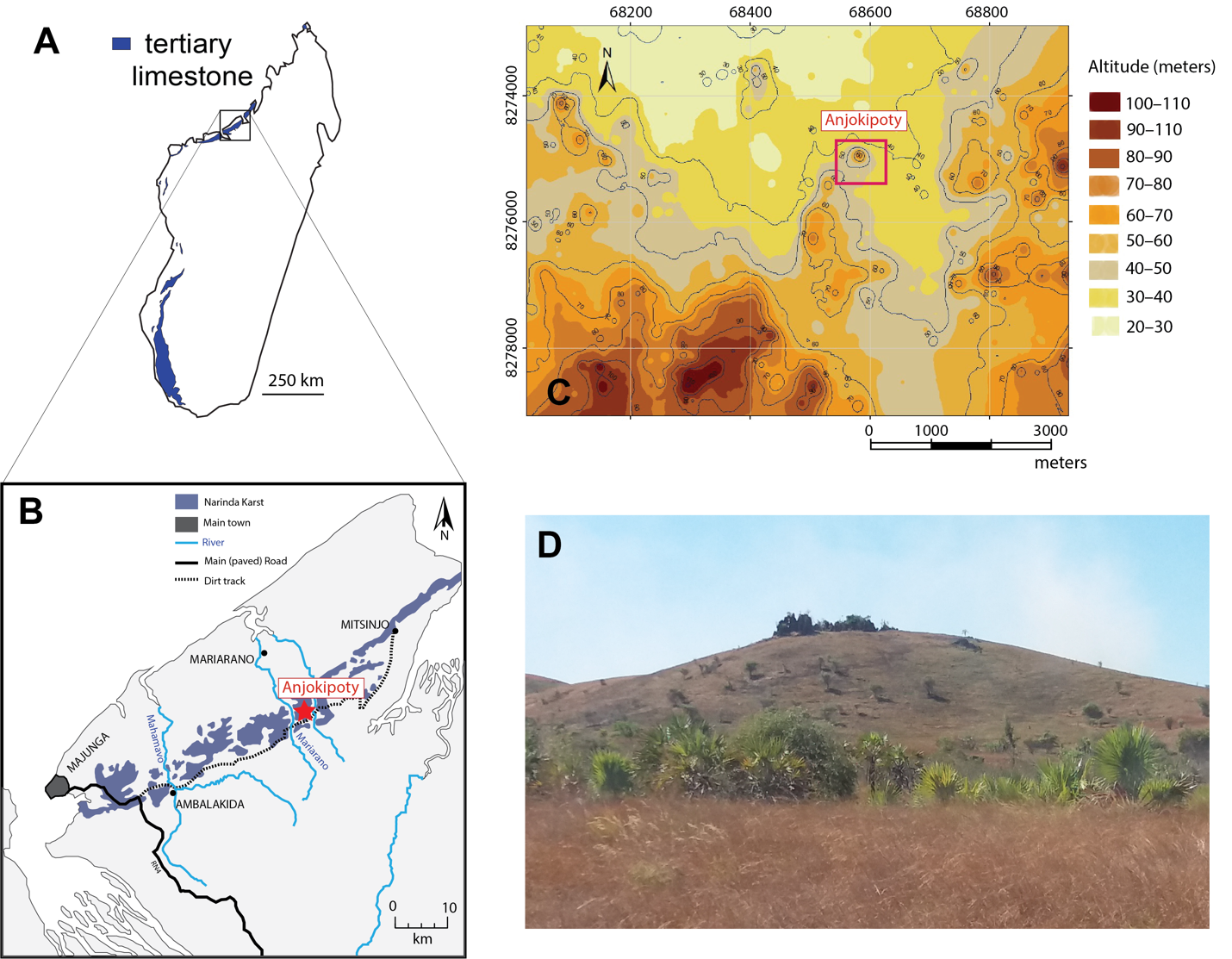


**Figure S1:** **Geological, geographical, and environmental settings of the study site**. (a) Map of Madagascar showing the extent of the Tertiary host carbonate outcrop where the Narinda karst develops (in blue). The study area is indicated with a black square. (b) Simplified map showing the southwestern part of the Narinda karst (blue) and the location of Anjokipoty Cave (red star). Figures S1a and S1b were modified from Voarintsoa et al.^1^ using Adobe Illustrator 26.3.1. These maps were digitized using the simplified geology map of Du Puy and Moat^2^ that was originally derived from Rakotobe and Ravalison^3^. (c) Elevation map of the surrounding area of Anjokipoty developed using the United States Geological Survey (USGS) Explorer Advanced Spaceborne Thermal Emission and Reflection Radiometer (ASTER) Global Digital Elevation Model (GDEM). This elevation map was georeferenced using the Oblique Mercator Laborde projection^4^. ASTER GDEM is a product of METI (Ministry of Economy, Trade, and Industry) and NASA (National Aeronautics and Space Administration). (d) Common hills and vegetation (C_4_ savanna and satra palms) cover surrounding the cave.

# S2. Cave monitoring at Anjokipoty Cave

Cave monitoring inside Anjokipoty Cave in July 2018 consists of setting up automatic logging of cave pCO_2_, relative humidity (RH), and air temperature using a 1% CO_2_ + RH/T Data Logger, which is equipped with a rechargeable lithium-ion battery pack (ref. CM-0212 from CO2meter.com). Details about the logger can be found in Voarintsoa et al.^5^. The cave atmophsere logging was set hourly to detect potential diurnal variations. The monitoring only covers a period of 10 days due to logger failure. Regardless, such information was valuable to assess the short term ventillation regime in the cave.

This short monitoring suggests that Anjokipoty is a well-ventilated cave and exhibit a strong diurnal variation in temperature and pCO_2_ (Figure S2). The average temperature inside the cave is 21°C, and it varies between 20°C (at night) and 22°C (during the day). The cave pCO_2_ varies between 482 and 729 ppm, with an average of 600 ppm during the monitoring period, and it shows similar diurnal variations as does the temperature. For relative humidity, the cave is humid with a near-saturated atmosphere (>95% relative humidity; Figure S3).


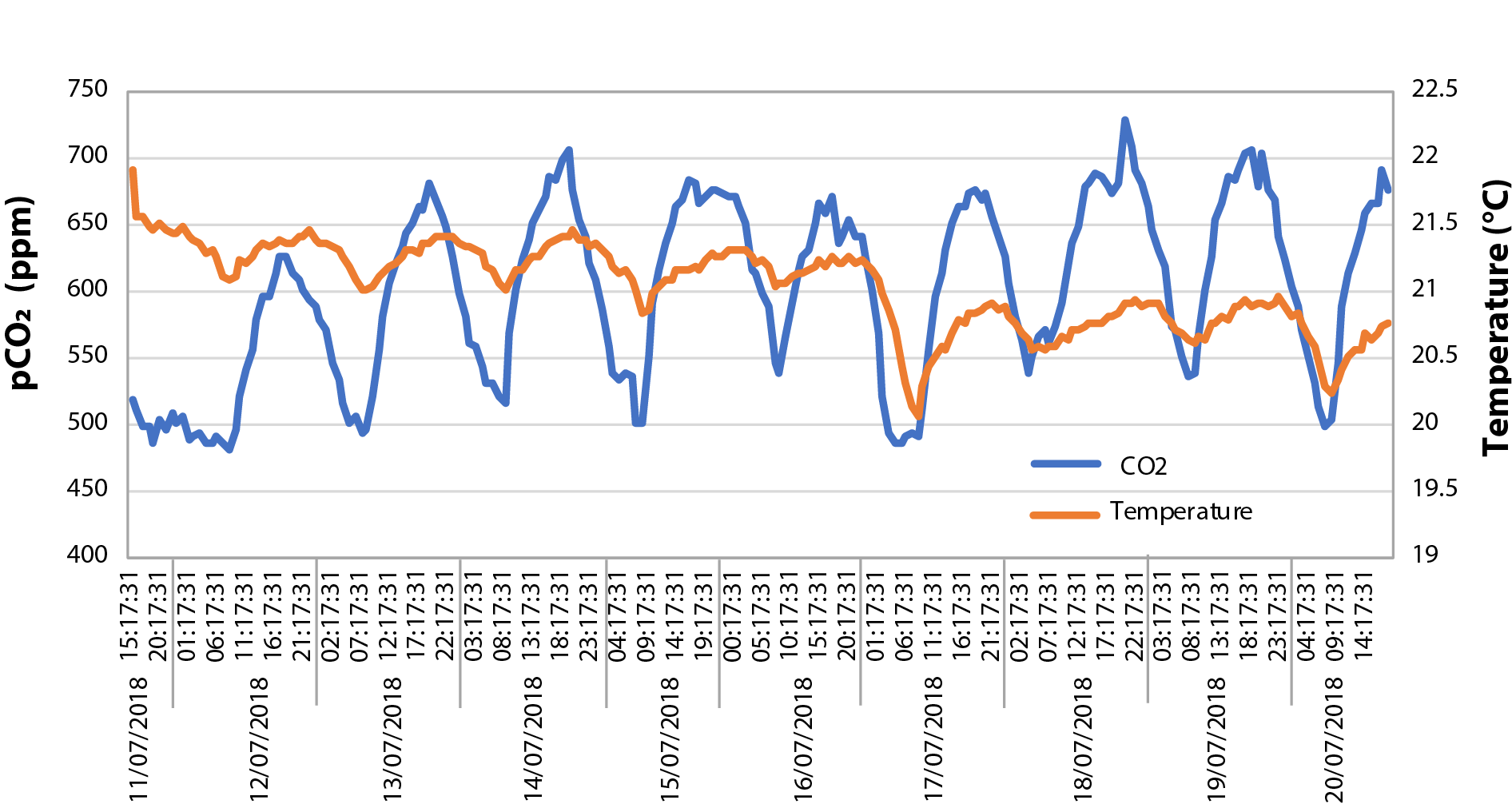


**Figure S2.** Variation of the cave pCO_2_ and temperature during a short continuous hourly monitoring in Anjokipoty Cave (between July 11 and July 20, 2018)


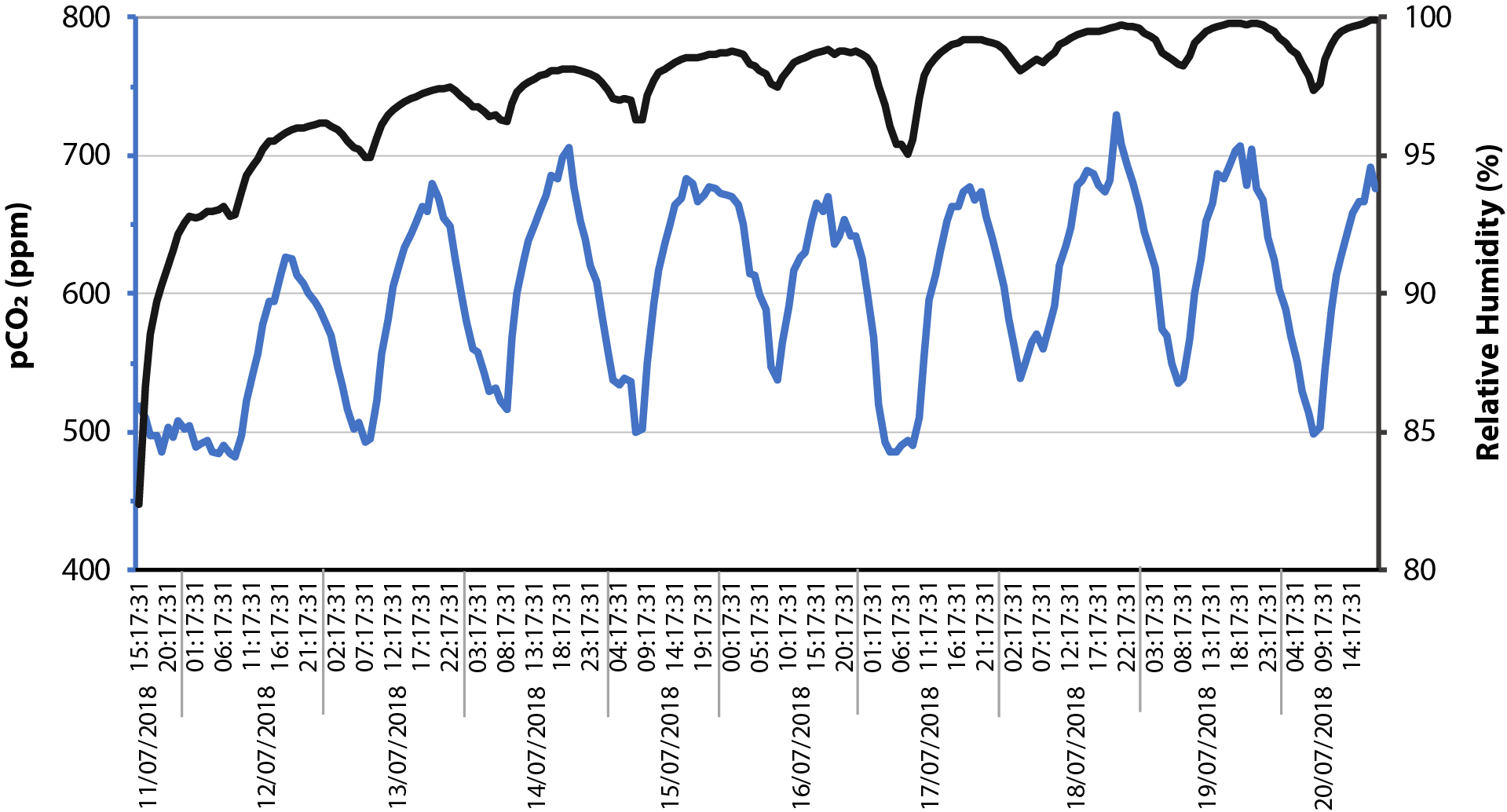


**Figure S3.** Variation of the cave CO_2_ and relative humidity (RH) during a short continuous hourly monitoring in Anjokipoty Cave (between July 11 and July 20, 2018). Note that the low RH at the beginning of the measurement indicates some needed time for the instrument to calibrate with the internal cave RH.

Drip waters from Anjokipoty caves were also collected in July (n=2) and September of 2018 (n=2) and April (n=2) and June of 2019 (n=4). Samplings and analytical procedures are similar to those described in Voarintsoa et al.^5^. The δ^18^O_w_ values of these drip water data along with the predicted stalagmite δ^18^O_c_ using cave analog experiment and natural cave isotopic fractionations equations are provided in Table S1 below.

# Table S1. Stable oxygen isotope values from Anjokipoty Cave drip water.

| **Date of sample collection** | **δ^18^O_w_**  **(‰, vs. VSMOW)** | **Cave analog Predicted δ^18^O_c_ values**^6^  **(‰, vs. VPDB)** | | **Natural Cave Predicted δ^18^O_c_ values**^7^  **(‰, vs. VPDB)** | |
| --- | --- | --- | --- | --- | --- |
| 7/11/18 1:22 PM | -4.07^a^ | -4.69 ^b^ | -5.61 ^c^ | -4.42 ^b^ | -5.37 ^c^ |
| 7/11/18 1:12 PM | -4.04 ^a^ | -4.66 ^b^ | -5.59 ^c^ | -4.40 ^b^ | -5.35 ^c^ |
| 9/12/18 11:50 AM | -3.01 ^a^ | -3.63 ^b^ | -4.56 ^c^ | -3.37 ^b^ | -4.32 ^c^ |
| 9/12/18 2:00 PM | -2.58 ^a^ | -3.21 ^b^ | -4.13 ^c^ | -2.94 ^b^ | -3.89 ^c^ |
| 4/18/19 1:26 PM | -3.77 ^a^ | -4.39 ^b^ | -5.32 ^c^ | -4.13 ^b^ | -5.08 ^c^ |
| 4/18/19 12:40 PM | -4.09 ^a^ | -4.71 ^b^ | -5.64 ^c^ | -4.45 ^b^ | -5.40 ^c^ |
| 6/10/19 1:47 PM | -3.35 ^a^ | -3.97 ^b^ | -4.90 ^c^ | -3.71 ^b^ | -4.66 ^c^ |
| 6/10/19 1:11 PM | -3.34 ^a^ | -3.96 ^b^ | -4.89 ^c^ | -3.70 ^b^ | -4.65 ^c^ |
| 6/10/19 1:15 PM | -3.50 ^a^ | -4.12 ^b^ | -5.05 ^c^ | -3.86 ^b^ | -4.81 ^c^ |
| 6/10/19 2:49 PM | -3.19 ^a^ | -3.82 ^b^ | -4.75 ^c^ | -3.55 ^b^ | -4.50 ^c^ |
| **Average** | **-3.49** | **-3.49** | **-5.04** | **-3.49** | **-4.80** |
| **stdev** | **0.5** | **0.5** | **0.50** | **0.5** | **0.50** |

^a^ Measured drip water δ^18^O from Anjokipoty caves.

^b^ Predicted carbonates δ^18^O (assuming that mineralogy is calcite) using the short-term cave climate dataset in winter (see above).

^c^ Predicted carbonates δ^18^O (assuming that mineralogy is calcite) using the mean annual 2018–2019 temperature of the region in Mahajanga^5^.

# S3. Sample description


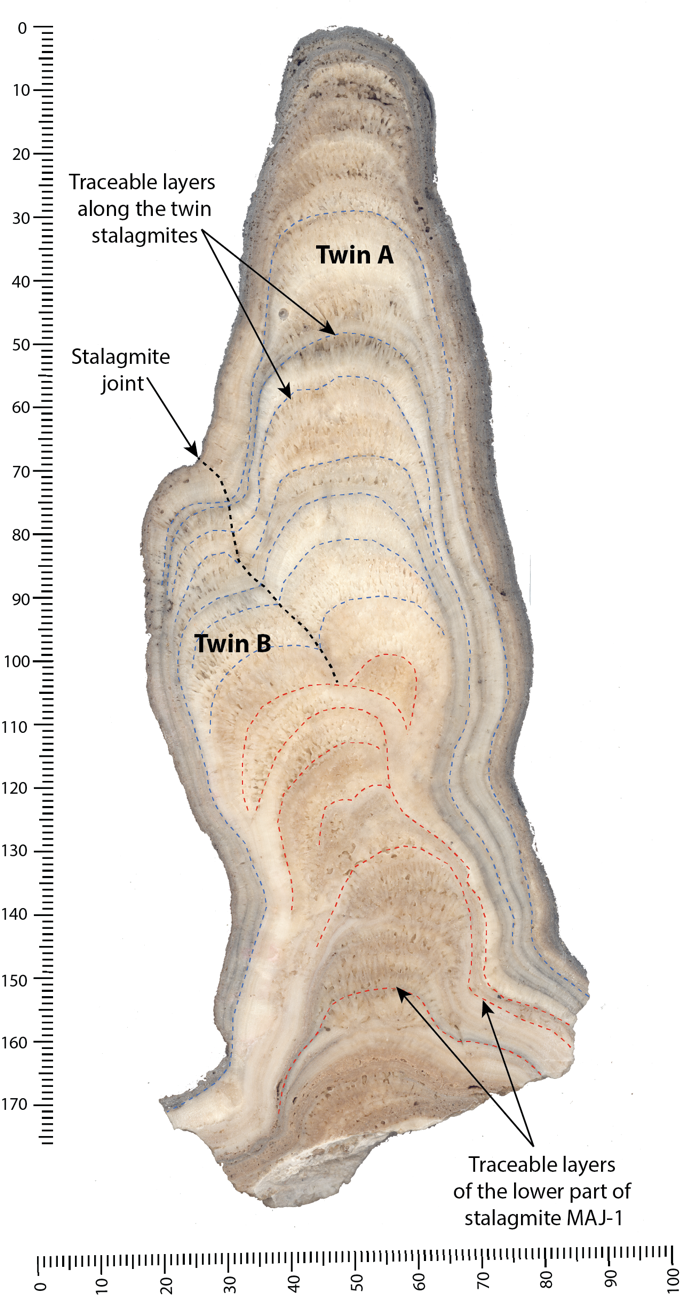


**Figure S4.** Image of Stalagmite MAJ-1 showing the two twins at the upper 110mm, that are separated by a joint (dashed black line). Traceable spelean layers from the lower part of this stalagmite are indicated in thin dashed red lines, and the continuous traceable layers between Twin A and Twin B are indicated in thin dashed blue lines.

# S4. Radiometric dating

## S4.1. StalAge Monte Carlo simulations

The age model of Stalagmite MAJ-1 was created with StalAge in R 2.10.1 using the code of Scholz and Hoffmann^8^. StalAge is an algorithm that uses Monte-Carlo simulations to develop the best fit chronology from stratigraphically ordered U-Th dataset to estimate the age of each sample along the growth axis of the stalagmite. We used the five U-Th dataset (Table S2) and individual sampling locations of both stable isotopes and elemental traces from LA-ICP-MS, by recording the distance from top (dft). To create the age model, the original U-Th dataset (expressed in BP with the corresponding 2sigma errors) was imported to R and then plotted (Figure S5) to further run a Monte-Carlo simulation (Figure S6). The entire dataset was then screened for major and minor outliers (Figure S7), to finally produce the best fit and the 95% confidence interval (Figures S8-S9) using the locations of the stable isotopes and trace elements (dft).

**Figure S5.** Original age data from U-Th dataset.

**Figure S6.** Monte-Carlo simulation for age modeling in StalAge.

**Figure S7.** Dataset screened for major and minor outliers.

**Figure S8.** Final age model with screened errors created using StalAge.

**Figure S9.** Final age model with original errors.

## S3.2. Radiometric results

# Table S2. ^230^Th dating results for Stalagmite MAJ-1. The error is 2σ.

^230^Th dating results.  The error is 2$\sigma$.

^*^ δ^234^U = ([^234^U/^238^U]_activity_-1) x 1000.

^**^ δ^234^U_initial_ was calculated based on ^230^Th age (T), that is, δ^234^U_initial_ = δ^234^U_measured_ x e^𝜆234xT^.

^***^BP stands for ‘Before Present’ where the ‘Present’ is defined as the year AD 1950.

# S5. Mosaic of microphotographs of Stalagmite MAJ-1

**Figure S10.** Stalagmite MAJ-1and various sampling locations. a) The entire Stalagmite MAJ-1 slabbed with thick section locations outlined. b) Thick sections with LA-ICP-MS line scan locations (red lines).


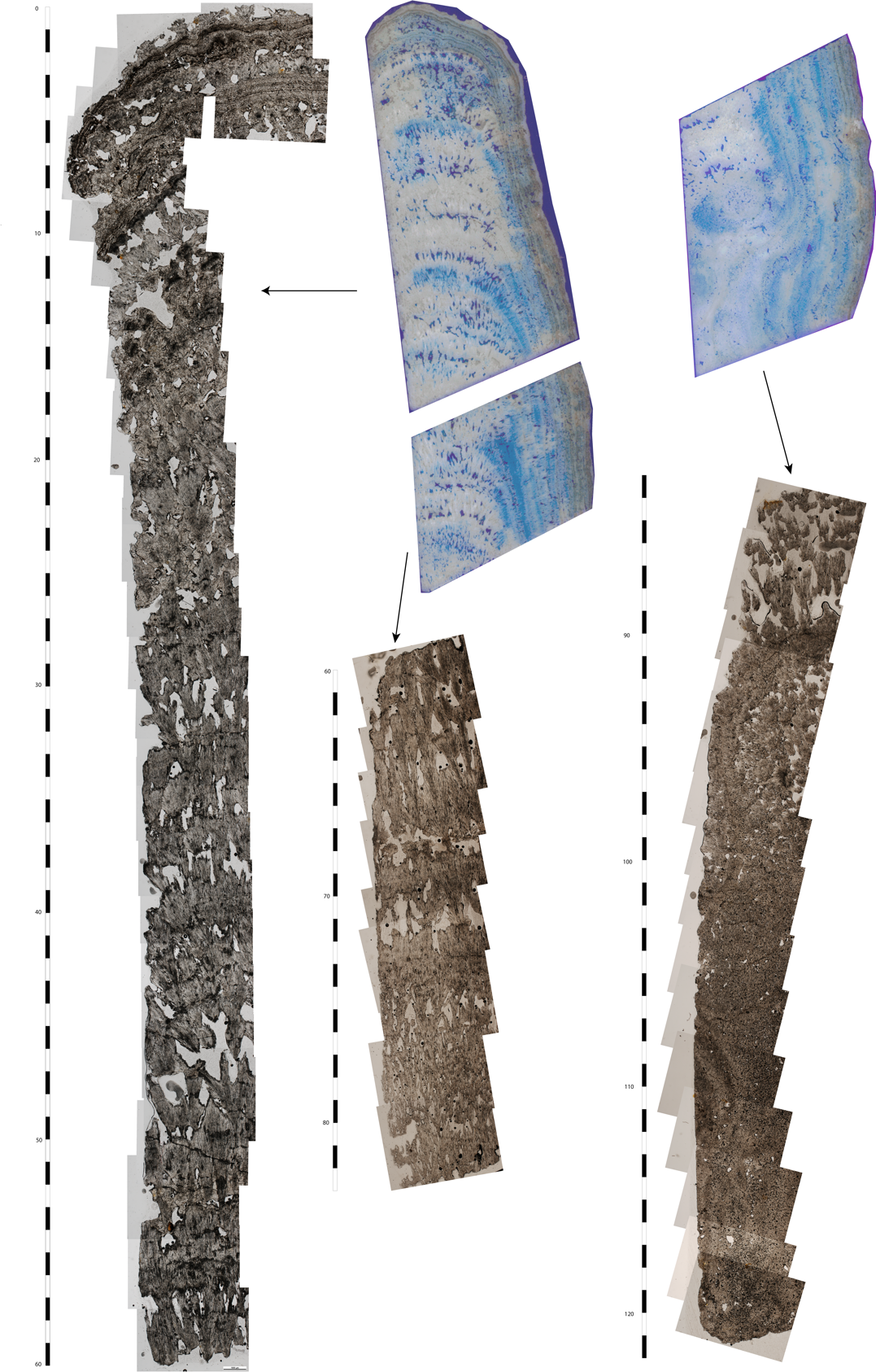


**Figure S11.** Mosaic of a suite of microphotographs taken from Stalagmite MAJ-1 thin sections. Note that the black and white stripes represent the 1mm-interval.

# S6. More details about mineralogy


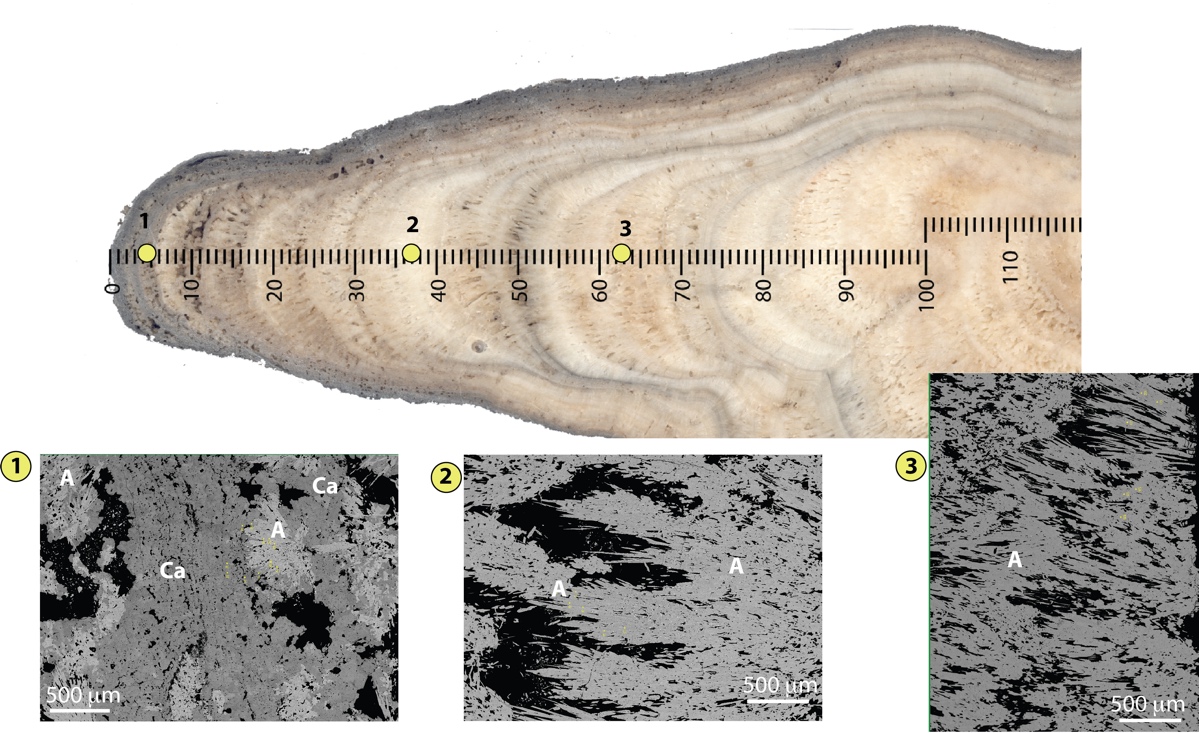


**Figure S12.** Image of the upper part of Stalagmite MAJ-1 (upper figure) and relevant BSE images (lower figure) illustrating the various mineralogy. Calcite (Ca) and Aragonite (A). Also note the change to darker color at the upper part of the stalagmite.


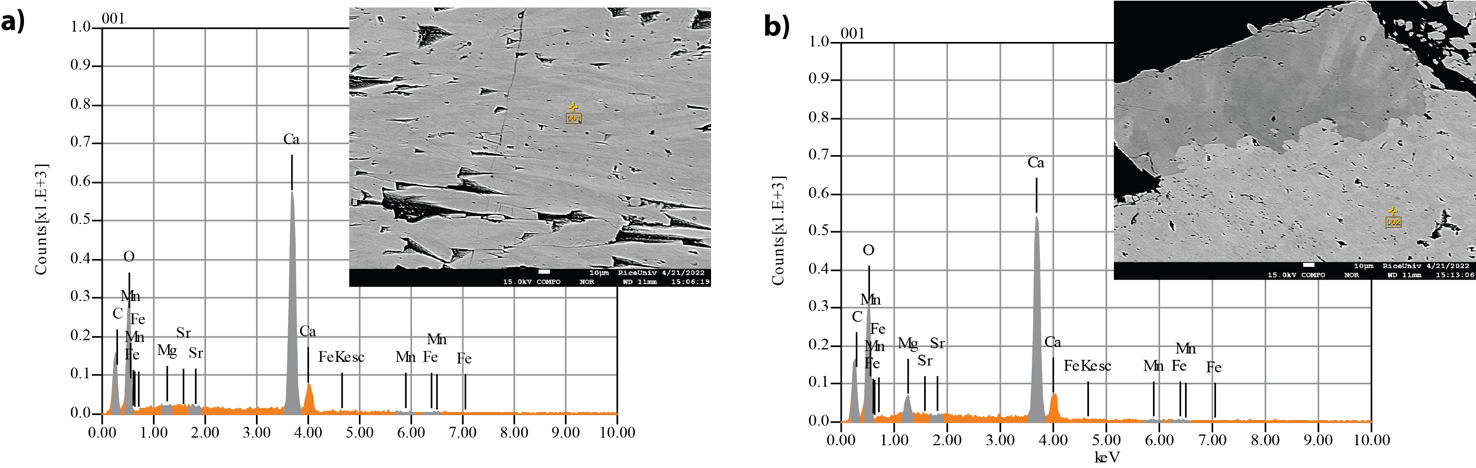


**Figure S13.** BSE images of CaCO_3_ polymorphs along with their respective EDS pattern found in Stalagmite MAJ-1. a) Aragonite. Note the acicular/columnar fabric. b) Mg calcite. Note the rhombic structure of calcite and the Mg peak in the EDS pattern.


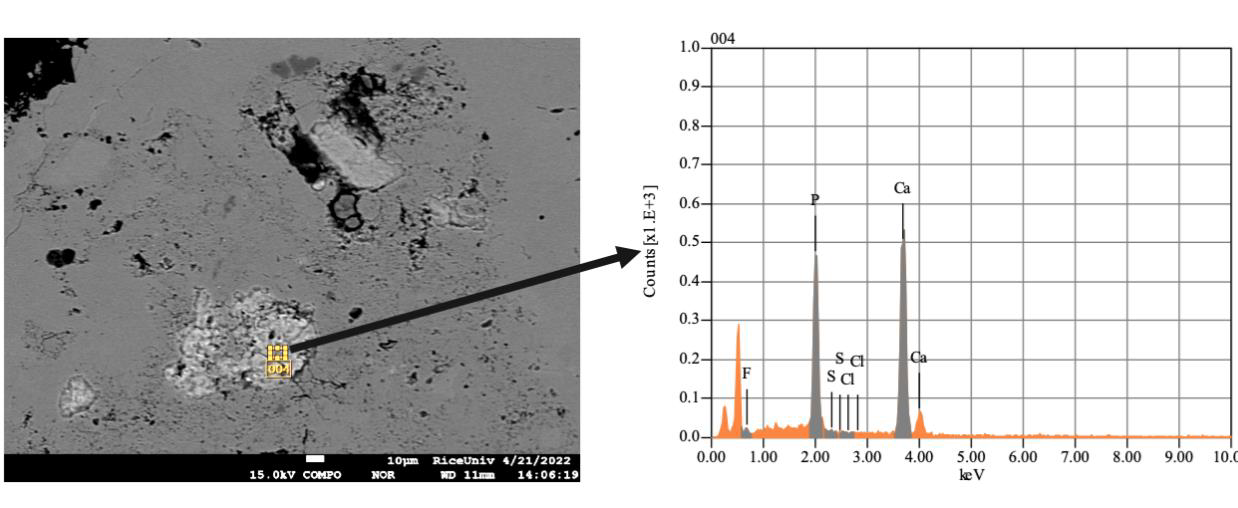


**Figure S14.** BSE image of apatite (left) and associated EDS pattern (right). Location of EDS analysis is given by the black arrow. Dark grey surrounding fluorapatite is calcite.

# S7. A review of stable isotopes variations in stalagmite

## Carbon isotopes

The stable isotope of carbon composition of stalagmites (δ^13^C_c_) may vary depending on the changes of three main reservoirs, including 1) the CO_2_ composition in the atmosphere ^9–11^; 2) the extent and nature of vegetation cover, with a higher δ^13^C_c_ values under C_4_ plants coverage ^12,13^, which is the dominant type of vegetation in the region, and the carbon isotopic composition of the host carbonate rock, known to have varied over geologic time ^14^. Besides variations within these reservoirs, it was reported that the residence time of the dissolved inorganic carbon, DIC, in the epikarst above the cave, as well as a precipitation of carbonates prior to reaching the apex of the stalagmite, aka prior calcite precipitation (PCP) and prior aragonite precipitation (PAP), can significantly impact the δ^13^C_c_ of the stalagmites ^15–18^. In a parallel monitoring effort to understand the environmental and climatic transfer to caves in the nearby Anjohibe Cave, Voarintsoa et al.^5^ also found that degassing led to an increase in δ^13^C of the DIC, the value of which is paralleled in the precipitated carbonate with an isotopic fractionation value (1000ln^13^α_Cc-DIC_) of ~2–3 between the precipitated carbonate (Cc) and the DIC, with ^13^α =(δ^13^C_Cc_, VPDB + 1000)/(δ^13^C_DIC_, VPDB + 1000). Those relationships and factors are important in understanding δ^13^C variations in stalagmites.

## Oxygen isotopes

The stable isotope of oxygen in stalagmites (δ^18^O_c_) are closely controlled by the δ^18^O values of the cave drip water and the temperature inside the cave, as demonstrated by the linear relationship between the calcite–water isotopic fractionation factor and cave temperature from several locations worldwide, 1000 ln^18^α= 16.9±0.6 (10^3^/T(°K) – 27.4±2.1 (e.g., ^19–21^). Cave temperature commonly represents the mean annual temperature of the surrounding environment (e.g., ^5,22–24^) and is expected to vary less at shorter time scale (e.g., ^23,25^). Drip water δ^18^O, in contrast, is more variable especially if the sourcing rainfall that supplies water to the cave changes, either during raindrop evaporation/re-evaporation ^26–28^, changes in the rainfall sourcing vapor^29–31^, changes in the distance of transport from the source, changes in the amount of precipitation^32–34^, or changes in the atmospheric temperature during rainfall. Those factors are reviewed in McDermott^35^, in Lachniet^36^, and in Wong and Breecker ^37^ for global speleothems, and in Voarintsoa^21^ for Madagascar in particular.

# S8. Sea surface temperature teleconnection

More work is still needed to understand the decadal/multidecadal variations of climate in northwestern Madagascar. Below is a preliminary correlation test using composite coral records from Ifaty^38^, reconstructing the sea surface temperature anomaly in the Agulhas current, an important current in southwestern Madagascar that is tied with the Atlantic Meridional Overturning circulation, and the stalagmite δ^18^O records from Anjokipoty.


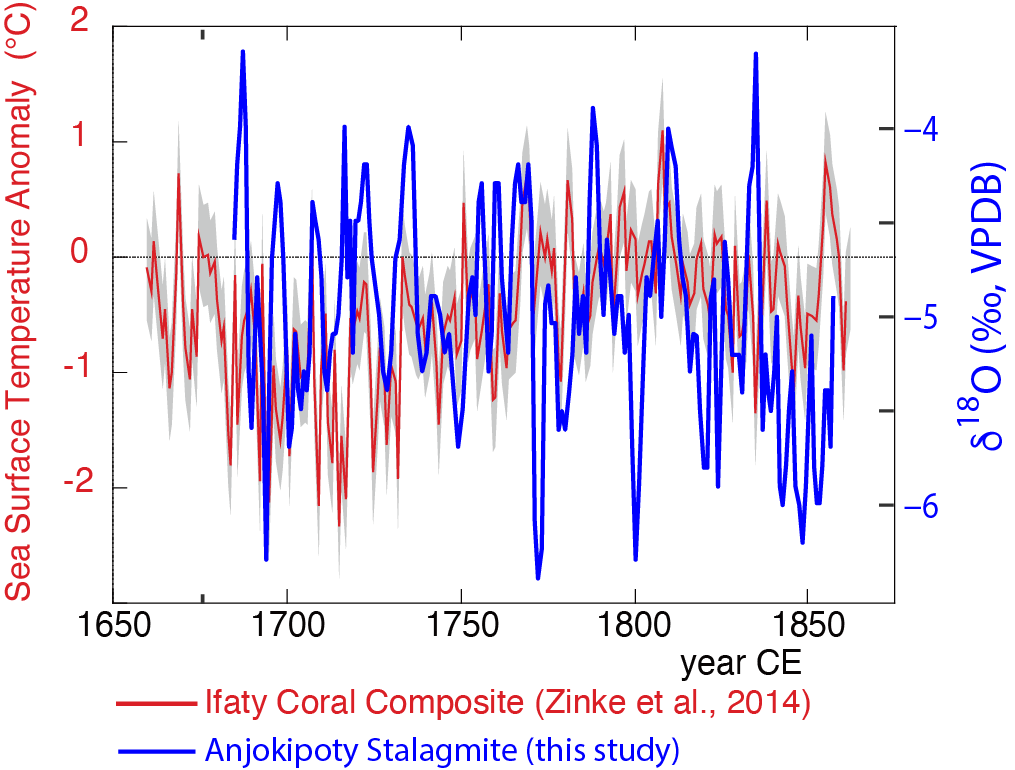


**Figure S15.** Sea surface temperature anomaly reconstruction for the Agulhas Current region using coral composite from Ifaty, in southwestern Madagascar (grey shading shows 2 standard error)^38^ correlated with δ^18^O records from Anjokipoty Cave, northwestern Madagascar (this study).

# S9. A review of uranium chemistry

Uranium is a naturally occurring radionuclide which has three isotopes namely ^238^U, ^235^U and ^234^U, with an abundance of 99.27, 0.72, and 0.005 %, respectively. Their respective radiological half-lives are 4.5 x 10^9^, 7.1 x 10^8^ and 2.5 x 10^5^ years and their respective activity percentage is 48, 2, and 48% (see an overview in Tripathi et al.^39^). Among these radionuclides, ^238^U and ^235^U are parent radionuclides of two natural occurring radioactive series, and disequilibrium between ^234^U and ^238^U in natural waters is very common^40,41^. Previous research has shown that dissolution of limestone results in a small (^234^U)/(^238^U) increase in water, which was assumed to result from the mobilization of uranium from minerals, the surface of which were previously depleted in ^234^U by the alpha recoil process (e.g., ^39,42–44^). With such hydrodynamic concept, researchers have used stalagmite (^234^U)/(^238^U) as a hydrology proxy (e.g.,^16,24,45^).

Uranium has a unique elemental and ionic properties^46^ with U^4+^ being generally immobile and U^6+^ mobile and conservative in oxidizing environment. In other words, U^6+^ is readily mobilized in the meteoric environment, as a highly soluble uranyl ion (UO_2_^2+^) and combine with other soluble anion such as CO_3_^2-^ to form carbonate complexes (e.g., UO_2_(CO_3_)_3_) that are commonly found in groundwaters (e.g.,^47–49^). The dissolved and mobile uranyl ion gets easily adsorbed by organic matter, such as fulvic and humic acid, inorganic colloids, and iron oxide in the absence of complexing elements, such as CO_3_^2-43,50^. Thus, U concentration in groundwater closely reflects redox conditions, so that higher U contents are associated with reduced conditions, due to the high organic content reducing U^6+^ to U^4+^ (e.g.^50^), while lower U contents are associated with oxidizing conditions, due to the strong mobility of UO_2_^2+^.

# S10. Biologic traces in Stalagmite MAJ-1


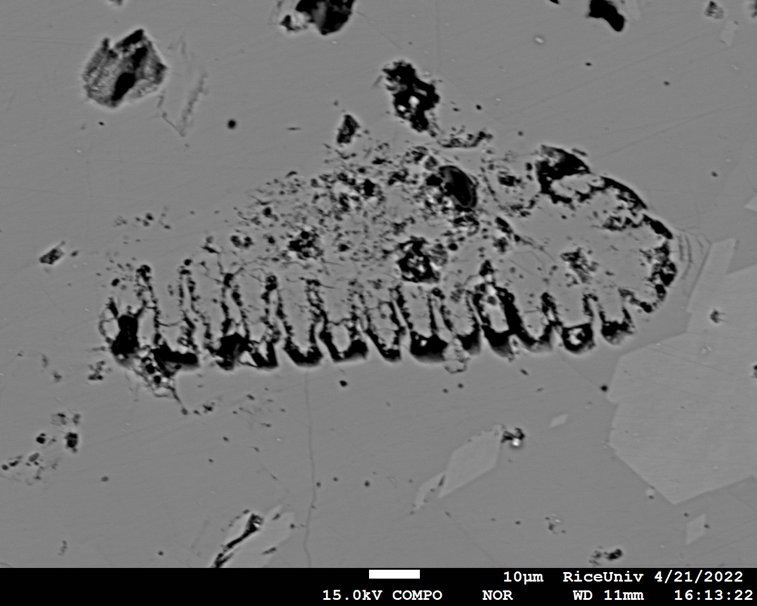


**Figure S16:** BSE image of a micro-organism observed at the upper micritic part of Stalagmite MAJ-1.


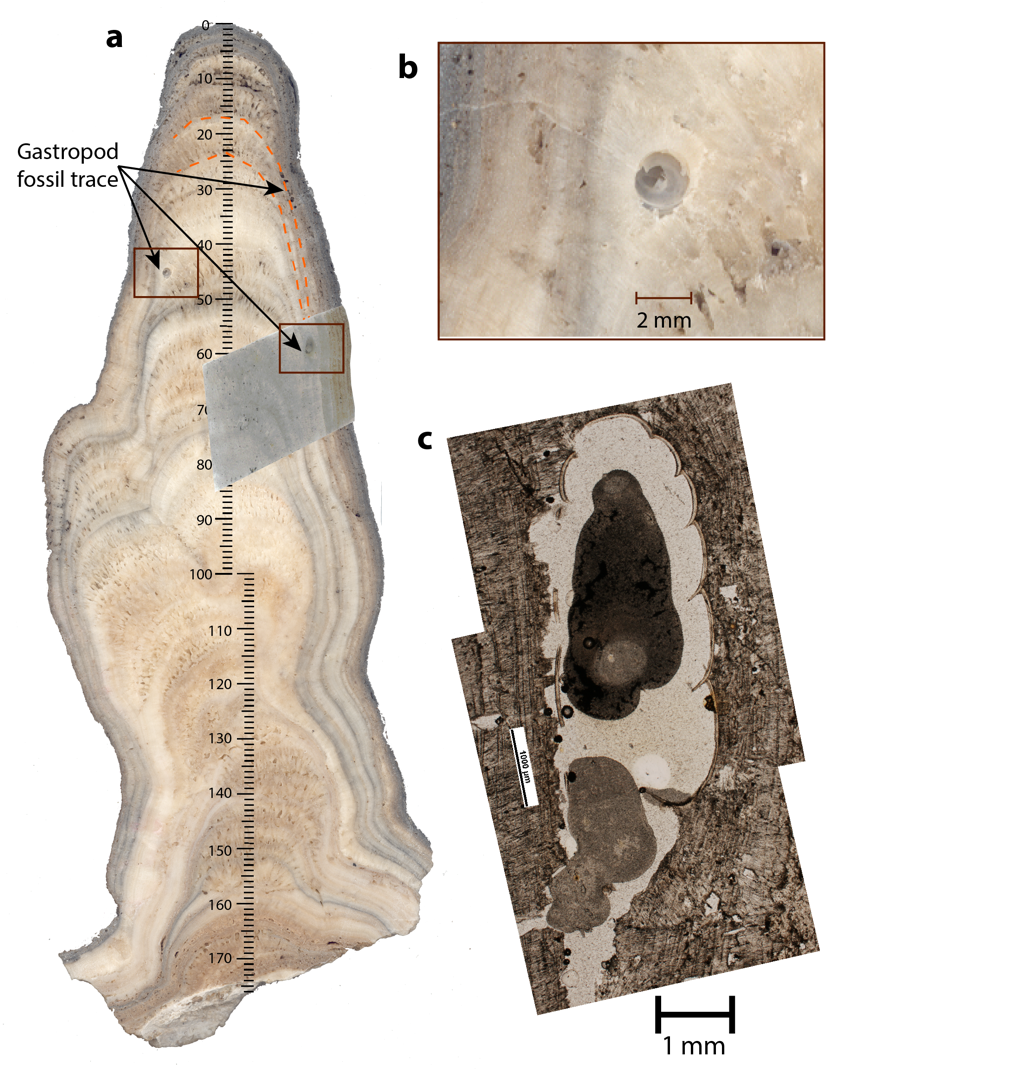


**Figure S17.** a) Stalagmite MAJ-1 with the location of the well-preserved gastropods traces. On its right side was overlain a photo of the middle regular thin section where the gastropod shell was found under the microscope. The dashed lines highlight the layer within which the gastropod shell was preserved. b) Close-up of the gastropod fossil trace preserved on the half of the stalagmite. c) Microphotograph the other gastropod fossil in its entirety showing no evidence of chemical alterations nor diagenesis.

# S11. Backscatter electron (BSE) microscopy.

Backscatter electron (BSE) microscopy was used to identify the sampling locations within the thick sections used for WDS analyses (Figure S18). BSE images highlight differences in elemental composition that allows for a better understanding of the mineralogy of the stalagmite (Figure 3). By analyzing the BSE images, it is possible to identify the different minerals present in the sample based on their brightness and morphology. Minerals with higher atomic numbers and densities, such as phosphates, will produce more backscattered electrons than minerals with lower atomic numbers and densities, such as carbonates. This results in a brightness contrast in the BSE image, with minerals of higher atomic number appearing brighter than minerals of lower atomic number. The chemistry of minerals identified in BSE were confirmed with energy dispersive spectroscopy (EDS). EDS works by detecting X-rays emitted by the sample when it is bombarded with high-energy electrons from the SEM’s electron beam. When the high-energy electrons interact with the sample, they can knock out electrons from the inner shells of the atoms in the sample, creating electron vacancies. Electrons from higher energy shells can then drop down to fill these vacancies, releasing energy in the form of X-rays with a characteristic energy that is unique to the element that emitted them. The detector then converts the X-rays into signals that are processed to provide a spectrum. This spectrum pattern is then used to identify the elements that are present in the sample. This process is very similar to WDS but has a higher spatial resolution. While WDS is more accurate, the analysis is time-consuming. EDS allows for a more rapid analysis. These two techniques for elemental analysis are complimentary to one another and this is why both techniques were used in the study.


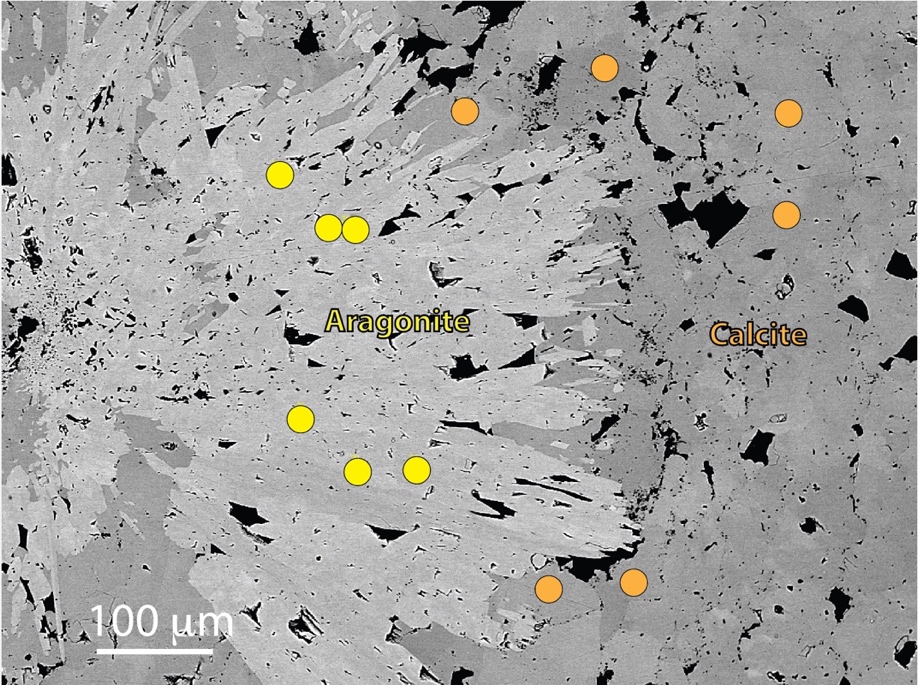


**Figure S18:** Back scattered Electron (BSE) image showing the locations of the EPMA measurements within calcite (orange dots) and aragonite (yellow dots) for the LA-ICP-MS matrix calibration (Equation 1, as discussed in the main manuscript).

# S12. Electron probe microanalysis (EPMA)

EPMA was performed at the Keith Weiss Geological Laboratory within Rice University using a JEOL JXA-8530F Field Emission Electron Probe Microanalyzer on 4/21/2022. The EPMA incident beam had a 1.5 *10^-7^ A probe current and a probe diameter of 10 µm. Elemental concentrations for Na, Mg, Si, P, S, Cl, Ca, Fe, Sr, Ba, Th, and U were collected with a Wavelength Dispersive Spectroscopy (WDS) system from several points along the growth axis of the two uppermost thick sections from Stalagmite MAJ-1. WDS reference materials used for calibration included calcite for Ca and Sr, plagioclase for Na, Ca, and Si, tugtupite for Na and Cl, olivine for Fe, Si, and Mg, anhydrite for S and Sr, apatite for P and Sr, barite for Ba, celestine for Sr, metals for Th and U, and a glass standard for P and S. Five spectrometers on the WDS system were simultaneously used to collect elemental data: TAP was used for Na and Si, TAP(2) for Mg and Sr, PETJ for Cl, Ca, and Th, LIFH for Fe, Ba, and Sr, and PETL for P, S, and U. A total of 194 spots were analyzed for MAJ-1. In WDS analysis a crystal is used to diffract and separate the X-rays emitted by the sample into specific wavelengths. Each wavelength corresponds to a specific characteristic X-ray line of an element. This allows for more precise elemental analysis. The collected data from Stalagmite MAJ-1 by WDS were used to determine the distribution and abundance of the elements in the sample.


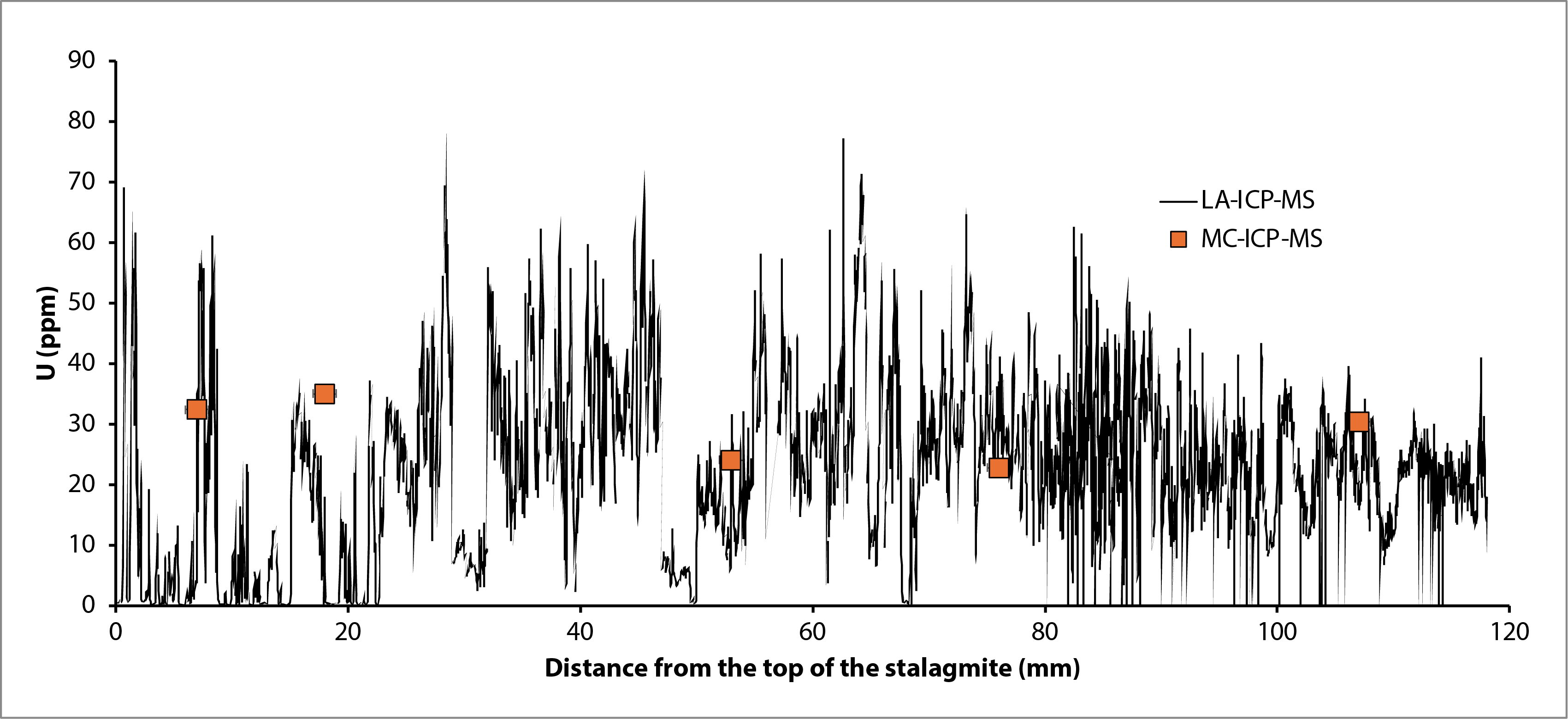


**Figure S19:** LA-IC-PMS concentration conversion verification using ICP-MS measured values from the U-Th dating (Table S2). Note that the marker size for the LA-ICP-MS data approximates the 1.5–2mm trench width, reflecting the average values for the LA-ICP-MS.

# References

1. Voarintsoa, N. R. G. *et al.* Three distinct Holocene intervals of stalagmite deposition and nondeposition revealed in NW Madagascar, and their paleoclimate implications. *Clim. Past* **13**, 1771–1790 (2017).

2. Du Puy, D. J. & Moat, J. A Refined Classification of the Primary Vegetation of Madagascar Based on the Underlying Geology: Using GIS to map its Distribution and to Assess its Conservation Status. *Biogéographie de Madagascar* 205–218 (1996).

3. Rakotobe, H. & Ravalison, P. Madagascar, Carte Géologique. Mise à jour en 1951 par Henri Besairie, Echelle au 1:1,000,000, trois feuilles en couleur. (1952).

4. Roggero, M. Laborde projection in Madagascar cartography and its recovery in WGS84 datum. *Appl Geomat* **1**, 131 (2009).

5. Voarintsoa, N. R. G., Ratovonanahary, A. L. J., Rakotovao, A. Z. M. & Bouillon, S. Understanding the linkage between regional climatology and cave geochemical parameters to calibrate speleothem proxies in Madagascar. *Science of The Total Environment* **784**, 147181 (2021).

6. Hansen, M., Scholz, D., Schöne, B. R. & Spötl, C. Simulating speleothem growth in the laboratory: Determination of the stable isotope fractionation (δ^13^C and δ^18^O) between H_2_O, DIC and CaCO_3_. *Chemical Geology* **509**, 20–44 (2019).

7. Voarintsoa, N. R. G. A 3.5-year rainfall isotope records from northwestern Madagascar featuring 17Oexcess and implication for paleoclimate research. *Applied Geochemistry* **184**, 106335 (2025).

8. Scholz, D. & Hoffmann, D. L. StalAge – An algorithm designed for construction of speleothem age models. *Quaternary Geochronology* **6**, 369–382 (2011).

9. Affek, H. P. & Yakir, D. The Stable Isotopic Composition of Atmospheric CO_2_. in *Treatise on Geochemistry* 179–212 (Elsevier, 2014). doi:10.1016/B978-0-08-095975-7.00407-1.

10. Broecker, W. S. & McGee, D. The ^13^C record for atmospheric CO_2_: What is it trying to tell us? *Earth and Planetary Science Letters* **368**, 175–182 (2013).

11. Verburg, P. The need to correct for the Suess effect in the application of δ^13^C in sediment of autotrophic Lake Tanganyika, as a productivity proxy in the Anthropocene. *J Paleolimnol* **37**, 591–602 (2007).

12. Ehleringer, J. R. & Cerling, T. E. C3 and C4 photosynthesis. *Encyclopedia of global environmental change* **2**, 186–190 (2002).

13. Gillson, L., Waldron, S. & Willis, K. J. Interpretation of soil δ^13^C as an indicator of vegetation change in African savannas. *J Vegetation Science* **15**, 339–350 (2004).

14. Saltzman, M., Thomas, E. & Gradstein, F. Carbon isotope stratigraphy. *The geologic time scale* **1**, 207–232 (2012).

15. Fohlmeister, J. *et al.* Main controls on the stable carbon isotope composition of speleothems. *Geochimica et Cosmochimica Acta* **279**, 67–87 (2020).

16. Jamieson, R. A. *et al.* Intra- and inter-annual uranium concentration variability in a Belizean stalagmite controlled by prior aragonite precipitation: A new tool for reconstructing hydro-climate using aragonitic speleothems. *Geochimica et Cosmochimica Acta* **190**, 332–346 (2016).

17. Treble, P. C. *et al.* Impacts of cave air ventilation and in-cave prior calcite precipitation on Golgotha Cave dripwater chemistry, southwest Australia. *Quaternary Science Reviews* **127**, 61–72 (2015).

18. Wassenburg, J. A. *et al.* Calcite Mg and Sr partition coefficients in cave environments: Implications for interpreting prior calcite precipitation in speleothems. *Geochimica et Cosmochimica Acta* **269**, 581–596 (2020).

19. Johnston, V. E., Borsato, A., Spötl, C., Frisia, S. & Miorandi, R. Stable isotopes in caves over altitudinal gradients: fractionation behaviour and inferences for speleothem sensitivity to climate change. *Clim. Past* **9**, 99–118 (2013).

20. Tremaine, D. M., Froelich, P. N. & Wang, Y. Speleothem calcite farmed in situ: Modern calibration of δ^18^O and δ^13^C paleoclimate proxies in a continuously-monitored natural cave system. *Geochimica et Cosmochimica Acta* **75**, 4929–4950 (2011).

21. Voarintsoa, N. R. G. The Malagasy monsoon over the Holocene: A review from speleothem δ^18^O_c_ records. *Malagasy Nature Special Issue* **16**, 56–78 (2021).

22. Baker, A. *et al.* Global analysis reveals climatic controls on the oxygen isotope composition of cave drip water. *Nat Commun* **10**, 2984 (2019).

23. Nava-Fernandez, C. *et al.* Pacific climate reflected in Waipuna Cave drip water hydrochemistry. *Hydrol. Earth Syst. Sci.* **24**, 3361–3380 (2020).

24. Oster, J. L., Montañez, I. P. & Kelley, N. P. Response of a modern cave system to large seasonal precipitation variability. *Geochimica et Cosmochimica Acta* **91**, 92–108 (2012).

25. Voarintsoa, N. R. G. *et al.* Multiple proxy analyses of a U/Th-dated stalagmite to reconstruct paleoenvironmental changes in northwestern Madagascar between 370 CE and 1300 CE. *Palaeogeography, Palaeoclimatology, Palaeoecology* **469**, 138–155 (2017).

26. Cuthbert, M. O. *et al.* Drip water isotopes in semi-arid karst: Implications for speleothem paleoclimatology. *Earth and Planetary Science Letters* **395**, 194–204 (2014).

27. Lee, J.-E. *et al.* Asian monsoon hydrometeorology from TES and SCIAMACHY water vapor isotope measurements and LMDZ simulations: Implications for speleothem climate record interpretation: Asian Monsoon Isotopes. *J. Geophys. Res.* **117**, D15112 (2012).

28. Markowska, M. *et al.* Modern speleothem oxygen isotope hydroclimate records in water-limited SE Australia. *Geochimica et Cosmochimica Acta* **270**, 431–448 (2020).

29. Affolter, S., Häuselmann, A. D., Fleitmann, D., Häuselmann, P. & Leuenberger, M. Triple isotope (δD, δ^17^O, δ^18^O) study on precipitation, drip water and speleothem fluid inclusions for a Western Central European cave (NW Switzerland). *Quaternary Science Reviews* **127**, 73–89 (2015).

30. Landais, A. *et al.* Combined measurements of ^17^O_excess_ and d-excess in African monsoon precipitation: Implications for evaluating convective parameterizations. *Earth and Planetary Science Letters* **298**, 104–112 (2010).

31. Sha, L. *et al.* Variations in triple oxygen isotope of speleothems from the Asian monsoon region reveal moisture sources over the past 300 years. *Commun Earth Environ* **4**, 384 (2023).

32. Bony, S., Risi, C. & Vimeux, F. Influence of convective processes on the isotopic composition (δ^18^O and δD) of precipitation and water vapor in the tropics: 1. Radiative-convective equilibrium and Tropical Ocean–Global Atmosphere–Coupled Ocean-Atmosphere Response Experiment (TOGA-COARE) simulations. *J. Geophys. Res.* **113**, D19305 (2008).

33. Dansgaard, W. Stable isotopes in precipitation. *Tellus* **16**, 436–468 (1964).

34. Risi, C., Bony, S. & Vimeux, F. Influence of convective processes on the isotopic composition (δ^18^O and δD) of precipitation and water vapor in the tropics: 2. Physical interpretation of the amount effect. *J. Geophys. Res.* **113**, D19306 (2008).

35. McDermott, F. Palaeoclimate reconstruction from stable isotope variations in speleothems: a review. *Quaternary Science Reviews* **23**, 901–918 (2004).

36. Lachniet, M. S. Climatic and environmental controls on speleothem oxygen-isotope values. *Quaternary Science Reviews* **28**, 412–432 (2009).

37. Wong, C. I. & Breecker, D. O. Advancements in the use of speleothems as climate archives. *Quaternary Science Reviews* **127**, 1–18 (2015).

38. Zinke, J., Loveday, B. R., Reason, C. J. C., Dullo, W.-C. & Kroon, D. Madagascar corals track sea surface temperature variability in the Agulhas Current core region over the past 334 years. *Sci Rep* **4**, 4393 (2014).

39. Tripathi, R. M. *et al.* Study of uranium isotopic composition in groundwater and deviation from secular equilibrium condition. *J Radioanal Nucl Chem* **295**, 1195–1200 (2013).

40. Fleischer, R. L. Alpha-recoil damage and solution effects in minerals: uranium isotopic disequilibrium and radon release. *Geochimica et Cosmochimica Acta* **46**, 2191–2201 (1982).

41. Thurber, D. L. Anomalous U ^234^ U ^238^ in nature. *J. Geophys. Res.* **67**, 4518–4520 (1962).

42. Bonotto, D. M. & Andrews, J. N. The mechanism of ^234^U^238^U activity ratio enhancement in karstic limestone groundwater. *Chemical Geology* **103**, 193–206 (1993).

43. Osmond, J. K. U-series nuclides as tracers in groundwater hydrology. in *Environmental Tracers in Subsurface Hydrology.* 145–174 (Kluwer Academic Publishers, Boston, 2000).

44. Osmond, J. K., Cowart, J. B. & Ivanovich, M. Uranium isotopic disequilibrium in ground water as an indicator of anomalies. *The International Journal of Applied Radiation and Isotopes* **34**, 283–308 (1983).

45. Zhou, J. *et al.* Geochemistry of speleothem records from southern Illinois: Development of (^234^U)/(^238^U) as a proxy for paleoprecipitation. *Chemical Geology* **221**, 1–20 (2005).

46. Railsback, L. B. An earth scientist’s periodic table of the elements and their ions. *Geol* **31**, 737 (2003).

47. Gayscone, M. Geochemistry of the actinides and their daughters. in *Uranium-Series Disequilibrium: Applications to Earth, Marine, and Environmental Sciences, 2nd Ed.* 34–61 (Clarendon Press, Oxford, 1992).

48. Grenthe, I. *et al.* Chemical Thermodynamics of Uranium. (1992).

49. Richards, D. A. & Dorale, J. A. Uranium-series Chronology and Environmental Applications of Speleothems. (2003) doi:https://doi.org/10.2113/0520407.

50. Brown, A. R. *et al.* The isotopic signature of UV during bacterial reduction. *Geochem. Persp. Let.* **29**, 45–50 (2024).
